# Molecular‐Metallic Binding Characteristics of the Intermetalloid f‐/p‐Block Cluster [(La@In_2_Bi_11_)_2_Bi_2_]^6−^

**DOI:** 10.1002/anie.202512019

**Published:** 2025-08-04

**Authors:** Harry Ramanantoanina, Julia Rienmüller, Yannick R. Lohse, Nina Rauwolf, Max Kehry, Cedric Reitz, Emily Marie Reynolds, Tim Prüßmann, Bianca Schacherl, Viktoriia A. Saveleva, Ruwini S. K. Ekanayake, Jörg Göttlicher, Bastian Weinert, Wim Klopper, Stefanie Dehnen, Tonya Vitova

**Affiliations:** ^1^ Institute for Nuclear Waste Disposal Karlsruhe Institute of Technology Kaiserstr. 12 76131 Karlsruhe Germany; ^2^ Institute of Nanotechnology Karlsruhe Institute of Technology Kaiserstr. 12 76131 Karlsruhe Germany; ^3^ Institute of Physical Chemistry Karlsruhe Institute of Technology Kaiserstr. 12 76131 Karlsruhe Germany; ^4^ European Synchrotron Radiation Facility (ESRF) 71, avenue des Martyrs, CS 40220 38043 Grenoble Cedex 9 France; ^5^ Institute for Photon Science and Synchrotron Radiation Karlsruhe Institute of Technology Kaiserstr. 12 76131 Karlsruhe Germany

**Keywords:** Covalency of Ln‐based bonds, GW‐BSE calculations, La HR‐XANES, La VB‐RIXS, Intermetallic clusters

## Abstract

Main goals of contemporary research in chemistry are to create new materials with unique properties and to understand the chemical bonding in them, especially between metal atoms in larger structures. The isolation of a single lanthanide atom in a In/Bi cage offers a non‐standard bonding situation, which deserves thorough exploration. In this study, the bonding behavior of La, In, and Bi atoms in the ternary cluster [(La@In_2_Bi_11_)_2_Bi_2_]^6−^ and the complex [La(C_5_Me_4_H)_3_] used for its synthesis are characterized and compared by applying high energy resolution X‐ray spectroscopy and computations. A clearly detectable covalent La(5d)─Bi(6p) interaction, induced in a highly electron‐rich environment, is illustrated. The electronic structure of the La atom can be described as having the character of an ion being trapped and bonded in a heterometallic Bi/In cage. The advanced X‐ray spectroscopic experimental tools applied here enable comparative studies of binding properties, focusing on different metals within the intermetalloid cluster. These tools can be employed iteratively to support the development of synthetic strategies that aim at tuning bond characteristics at the boundary of covalent and metallic bonding, thereby advancing the chemical and physical properties of novel multinary cluster compounds. The results were corroborated by *GW* and Bethe–Salpeter‐equation (*GW*‐BSE) calculations.

## Graphical abstract

A covalent interaction between La and Bi atoms in the intermetalloid f‐/p‐block cluster [(La@In_2_Bi_1__1_)_2_Bi_2_]^6–,^ similar to but larger than that in XeF_2_, is revealed based on quantum chemistry and valence band X‐ray emission spectroscopy.

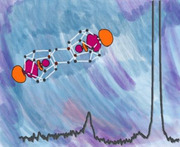

## Introduction

Compounds with intermetalloid clusters, in which an inner (interstitial) metal atom is surrounded by a shell of other (semi)metal atoms, have become a pronounced focus of chemical and materials research interest in the recent past.^[^ ^1^, ^2^, ^3^, ^4^, ^5^, ^6^, ^7^, ^8^, ^9^ ^]^ The aesthetics and structures of these clusters, together with uncommon bonding situations, have inspired experimental and theoretical work throughout. In particular, the goal is to gain a better understanding of the electronic properties of these nanomaterials, which can be viewed as models for cutouts of intermetallic phases and which show great potential in innovative materials development, for instance, as atomically‐precise catalysts.^[^ ^10^, ^11^ ^]^ Multimetallic clusters based on Bi atoms have been addressed in particular.^[^ ^12^ ^]^ Besides unique chemical features, they show great promise in exhibiting advanced magnetic, catalytic, or optical properties, which explains a growing body of literature describing success stories in this area.^[^ ^13^, ^14^ ^]^ However, there are still many open questions regarding Bi‐based clusters, ranging from formation mechanisms to stabilities and reactivities, which therefore require new experimental approaches to gain more sophisticated insight.^[^ ^12^, ^15^ ^]^

One of the unanswered fundamental questions concerns the nature of the interaction of interstitial atoms with the atoms of the cluster shell. This is particularly important for interstitial atoms from the lanthanide (Ln) series, as these are actively discussed as “dopants” for precise fine‐tuning the properties of intermetalloid clusters. However, this emerging combination of metals has only been fragmentarily researched to date.^[^ ^16^, ^17^ ^]^ We therefore aimed at gaining an in depth understanding of the electronic situation in a corresponding cluster with atomic precision. The cluster we chose for our study serves as a perfect molecular model to explore the degree of bond covalency, which ultimately affects the stability and reactivity of the clusters with incorporated lanthanide atoms. Herein, we take the first major step in this complex endeavor, specifically by revealing substantial covalent interaction between La and Bi through a combination of advanced experimental and theoretical approaches.

The compound that was selected for this study, [K(crypt‐222)]_6_[(La@In_2_Bi_11_)_2_Bi_2_]⋅3*en*⋅3tol (crypt‐222: 4,7,13,16,21,24‐hexaoxa‐1,10‐diazabicyclo[8.8.8]hexacosane, *en*: ethane‐1,2‐diamine, tol: toluene), is accessible in high yields and purity,^[^ ^18^ ^]^ which is not the case for other examples in the same way.^[^ ^18^ ^]^ The compound thus offers the opportunity to investigate the interaction between the involved metal atoms in more detail.

One of the most important means of tuning the interaction of interest is to control the composition, size, and shape of the clusters, which is a substantial challenge for synthetic chemists. It has been shown that organometallic Lewis acids like [Ln(C_5_Me_4_H)_3_] play an important role as reagents for the formation of Bi‐rich molecules, even without being necessarily involved in the final product.^[^ ^15^ ^]^ However, more often, the elements from the lanthanide series are incorporated into a 13‐ or 14‐atom main group metal shell. The cluster investigated herein, [(La@In_2_Bi_11_)_2_Bi_2_]^6−^, has so far been the one and only example of two identical 13‐atom clusters connected by two {*μ*‐Bi}^[^ ^19^ ^]^ bridges.^[^ ^16^, ^17^, ^18^, ^20^ ^]^

While the bonding situation has been investigated for many multimetallic clusters by computational studies, high energy resolution X‐ray absorption near edge structure (HR‐XANES) and valence band resonant inelastic X‐ray scattering (VB‐RIXS) methods have not yet been applied.^[^ ^21^ ^]^ The application of HR‐XANES and VB‐RIXS for electronic structure studies of Ln compounds is starting to gain momentum.^[^ ^22^ ^]^ We recently reviewed the bonding situation and evidence for bond covalency for lanthanides, emphasizing the potential and need to further develop and apply the Ln HR‐XANES/VB‐RIXS techniques for in depth characterization of the Ln–element bonding in unusual Ln materials.^[^ ^23^ ^]^

In this report, classical XANES as well as HR‐XANES and VB‐RIXS are applied to investigate the bonding properties from the perspective of the different atoms in the closed‐shell multimetallic cluster [(La@In_2_Bi_11_)_2_Bi_2_]^6−^, in order to obtain an overview of the binding interactions, specifically of the La atom with the cage Bi/In atoms. We corroborate the experimental studies with density functional theory (DFT) and *GW*‐based bonding analyses and calculations of La L_3_‐edge HR‐XANES spectra using the Bethe–Salpeter equation (BSE). In addition, we will address how the covalent bonding interactions between La and the surrounding metal atoms in [(La@In_2_Bi_11_)_2_Bi_2_]^6−^ compare with the situation in the closed‐shell [La(C_5_Me_4_H)_3_] reactant. For example, it was proposed that a U atom is much more readily incorporated in a Bi‐based multimetallic cluster than an atom of a lanthanide element, due to the stronger U–Bi covalent interactions, which drive the U incorporation when using [U(C_5_Me_4_H)_3_] as a reagent.^[^ ^15^ ^]^ Following up on this notion, we will gain understanding on the ability of La ions to form covalent bonds using 5d and 6s valence atomic orbitals in the named compounds.

## Results and Discussion

### Structure and Bonding Situation of the [(La@In_2_Bi_11_)_2_Bi_2_]^6−^ Cluster

**Figure 1:**
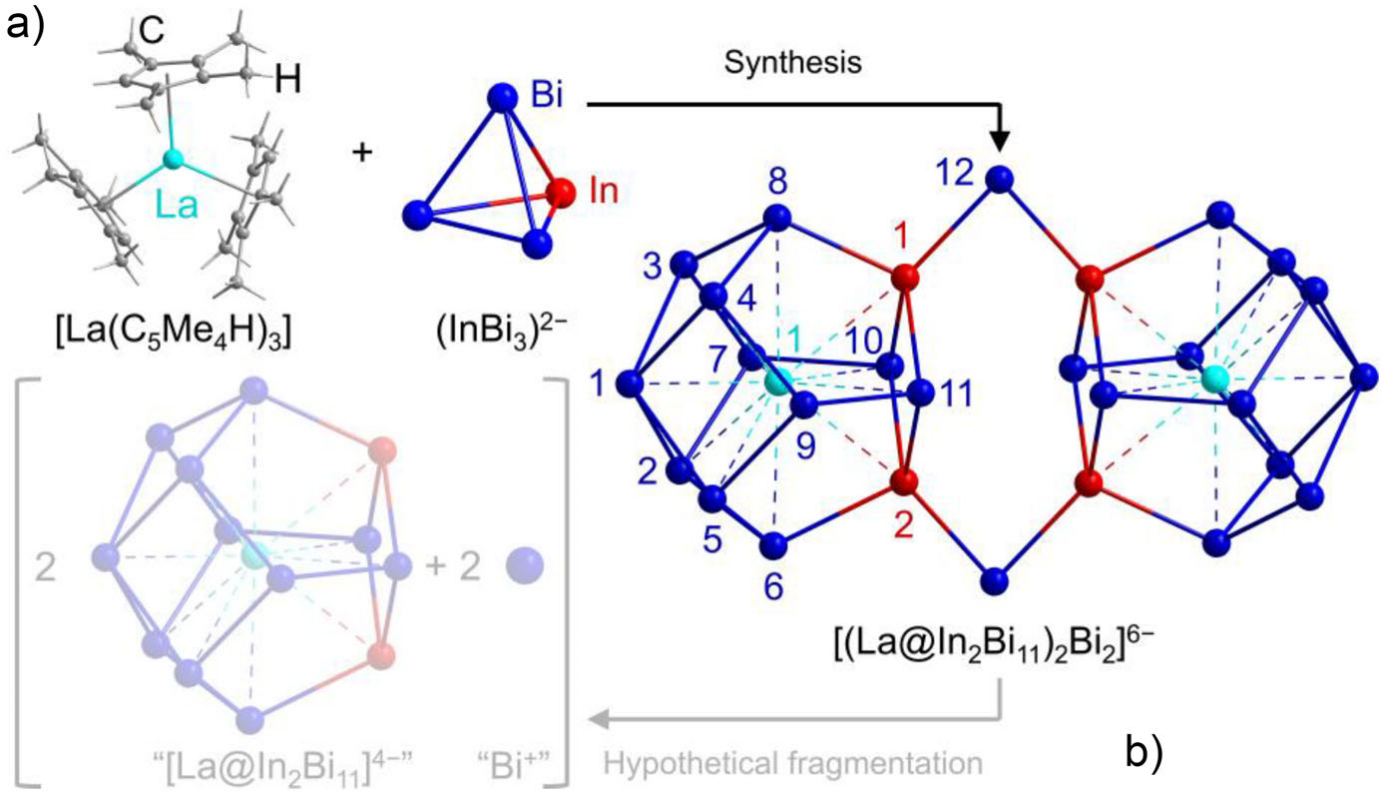
a) Schematic representation of the synthetic approach of [(La@In_2_Bi_11_)_2_Bi_2_]^6−^ by reaction of the precursor complex [La(C_5_Me_4_H)_3_] with the binary Zintl anion (InBi_3_)^2–^ (for details, see text). b) Hypothetic fragmentation (semi‐transparent mode) of the cluster into two monomeric units of the formula “[(La@In_2_Bi_11_)]^4−^” (analogous to the reported [(U@Tl_2_Bi_11_)]^4−[^ ^24^ ^]^) and two formerly bridging “Bi^+^” atoms, to illustrate the monomeric model compound studied computationally below; note that a real fragmentation would immediately consume “Bi^+^” in larger polybismuthide anions.

The synthesis and single‐crystal X‐ray diffraction (SC‐XRD) characterization of the [(La@In_2_Bi_11_)_2_Bi_2_]^6−^ anion, crystallizing with six equivalents of the counterion [K(crypt‐222)]^+^, three *en*, and three toluene units per formula unit in the salt [K(crypt‐222)]_6_[(La@In_2_Bi_11_)_2_Bi_2_]⋅3*en*⋅3tol, were done similarly as described previously^[^ ^18^ ^]^: [K(crypt‐222)]_6_[(La@In_2_Bi_11_)_2_Bi_2_]⋅3*en*⋅3tol was obtained by reacting a solution of [K(crypt‐222)]_2_(InBi_3_)⋅*en* and [La(C_5_Me_4_H)_3_] (cf. Figure 1a) in *en* after subsequent filtration and layering with toluene. After a few days, dark blueish‐black crystals of [K(crypt‐222)]_6_[(La@In_2_Bi_11_)_2_Bi_2_]⋅3*en*⋅3tol formed at the wall of the vessel. These crystals were washed with toluene and prepared into the sample holder for optical measurement using a spatula that was covered with immersion oil.^[^ ^18^ ^]^ The cluster anion, which can also be formulated as [(La@In_2_Bi_11_)(*μ*‐Bi)_2_(La@In_2_Bi_11_)]^6−^, consists of two nine‐faced polyhedra (enneahedra) comprising two indium and eleven bismuth atoms, which are connected by two additional μ‐bridging bismuth atoms. The cluster shells each enclose one lanthanum atom, which is located near the barycenter of the two enneahedra (cf. Figure 1b)

An {In_2_Bi_2_} ring serves as the basis for four condensed InBi_4_ pentagons. These, in turn, share two edges with four Bi_4_ vertices, with the latter meeting in an apical bismuth atom. The two enneahedra are linked by the two *μ*‐Bi bridges, which connect two indium atoms of neighboring cluster monomers each. The bonding situation was studied in detail with DFT, focusing on the Bi–In interactions in the bridge between the two clusters, making the double cluster more energetically stable than a single cluster.^[^ ^18^ ^]^ According to population analyses, the group of atoms numbered Bi1–Bi5 exhibits an overall three‐fold negative charge. The remaining two In and six Bi cluster shell atoms in each cage possess a total charge of 4−. However, it was shown that the larger Bi electronegativity within the covalent Bi–In interaction across the In–(*μ*‐Bi)–In bridges leads to electron transfer from (formal) In^2–^ to (formal) (*μ*‐Bi)^+^ to yield (formal) In^–^ and (formal) (*μ*‐Bi)^–^. As a consequence, each cluster half (including one of the *μ*‐Bi atoms) carries a total charge of 3–. It was concluded that the two La(III) atoms, located nearly in the barycenters of theenneahedra, have predominantly ionic interactions with the cage atoms.^[^ ^18^ ^]^ However, no detailed analysis of the La bonding situation was performed, and no experimental techniques sensitive to the La electronic structure and binding behavior were applied.

There are examples in the literature showing that Ln atoms can participate in covalent bonding, especially using their more spatially diffuse 5d orbitals, as compared to the more strongly localized 4f valence orbitals.^[^ ^22^, ^25^, ^26^, ^27^ ^]^ Applying high energy resolution X‐ray spectroscopy, we will explore in the following section whether the La 5d valence orbitals participate in covalent bonding and to what extent, specifically for the [(La@In_2_Bi_11_)_2_Bi_2_]^6−^ cluster and the [La(C_5_Me_4_H)_3_] reagent.

### Binding Properties of the La Atoms Probed by L_3_‐Edge VB‐RIXS, La L_3_‐Edge HR‐XANES, and DFT Calculations

**Figure 2:**
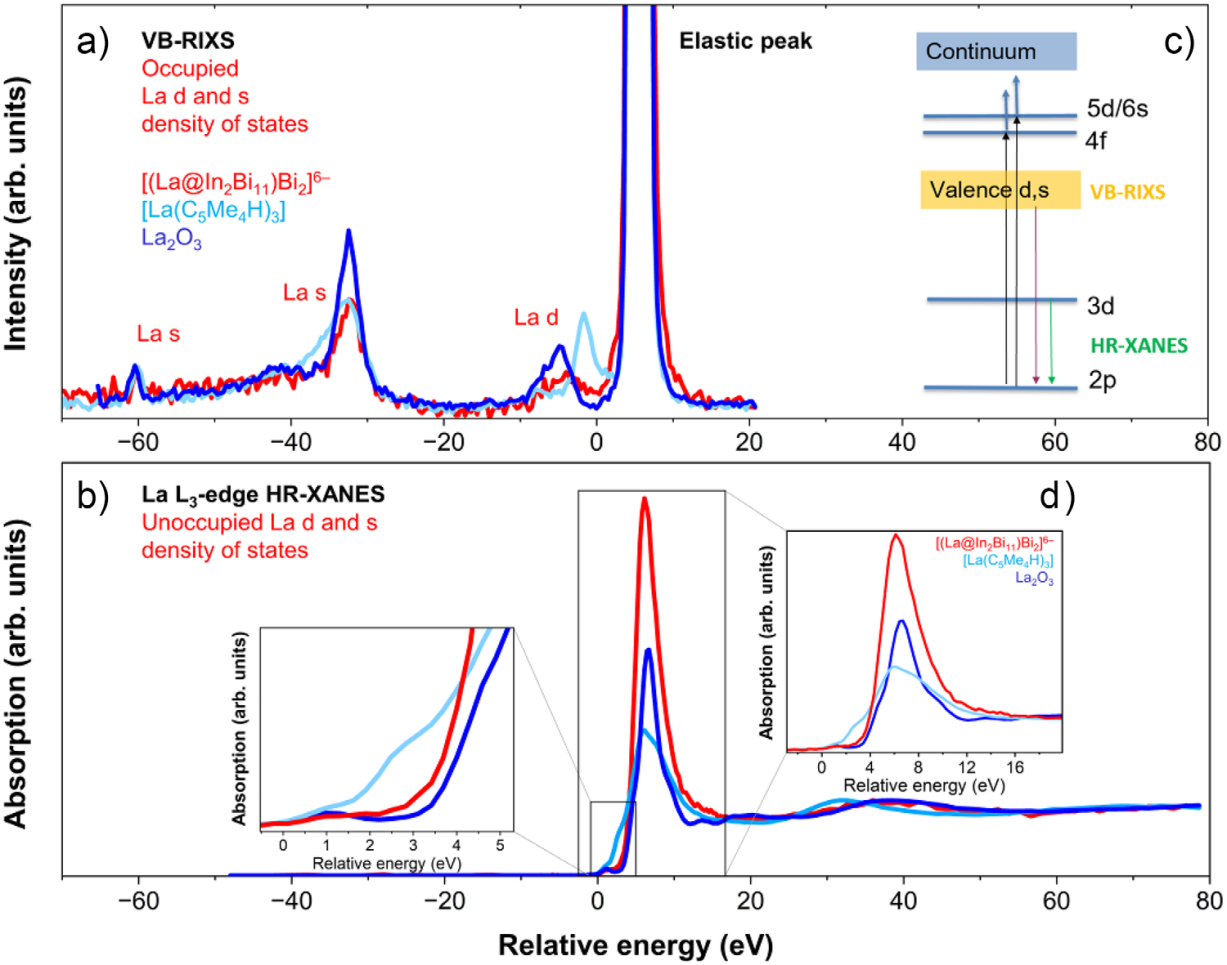
a) Normalized La L_3_‐edge VB‐RIXS, b) HR‐XANES spectra, c) the electron transitions involved, and (d) the pre‐edge and main absorption peaks of the La L_3_‐edge HR‐XANES spectra of [(La@In_2_Bi_11_)_2_Bi_2_]^6−^, [La(C_5_Me_4_H)_3_], and La_2_O_3_.

Figure 2 depicts the La L_3_‐edge VB‐RIXS (a) and HR‐XANES (b,d) spectra of [La(C_5_Me_4_H)_3_], [(La@In_2_Bi_11_)_2_Bi_2_]^6−^, and La_2_O_3_. They probe the unoccupied (HR‐XANES) and occupied (VB‐RIXS) La 5d and La 5s/6s density of states (DOS) in the presence of a core‐hole in the intermediate as well as the final state. Note that metallic La has a 5d^1^6s^2^ valence electron configuration, whereas the electron configuration of La(III) is 5d^0^6s^0^. Figure 2c describes the electron transitions involved in La L_3_‐edge HR‐XANES and VB‐RIXS governed by the electric‐dipole selection rules ∆*l* = ±1, ∆*J* = 0, ±1 for the 2p_3/2_ → 5d/6s excitation and the “3d (HR‐XANES)/La d and s in the valence band (VB‐RIXS)” → 2p_3/2_ deexcitation processes. Note that *l* is the orbital angular moment of a single electron, whereas *J* is the total angular moment of the atom participating in the excitation process.

Figure 2d illustrates the pre‐edge and main absorption peak (so‐called white line, WL) regions of the La L_3_‐edge HR‐XANES spectra of [La(C_5_Me_4_H)_3_], [(La@In_2_Bi_11_)_2_Bi_2_]^6−^, and La_2_O_3._ The pre‐edge peak at about 1 eV (see the inset of Figure 2b) is only resolved in the HR‐XANES spectra and occurs as a result of weak electric‐quadrupole transitions to 4f unoccupied states (2p_3/2_ → 4f), but it gains intensity when 4f and 5d are mixed in a non‐centrosymmetric structure.^[^ ^23^, ^28^, ^29^, ^30^, ^31^ ^]^ Since the probed 4f unoccupied states have bonding equivalents that are occupied, the pre‐edge is also an indirect probe for the mixture of 5d and 4f orbitals in the occupied valence band. There are clear differences between the intensity and shape of spectra of the La atoms included in the precursor [La(C_5_Me_4_H)_3_] or the cluster [(La@In_2_Bi_11_)_2_Bi_2_]^6−^.

**Figure 3:**
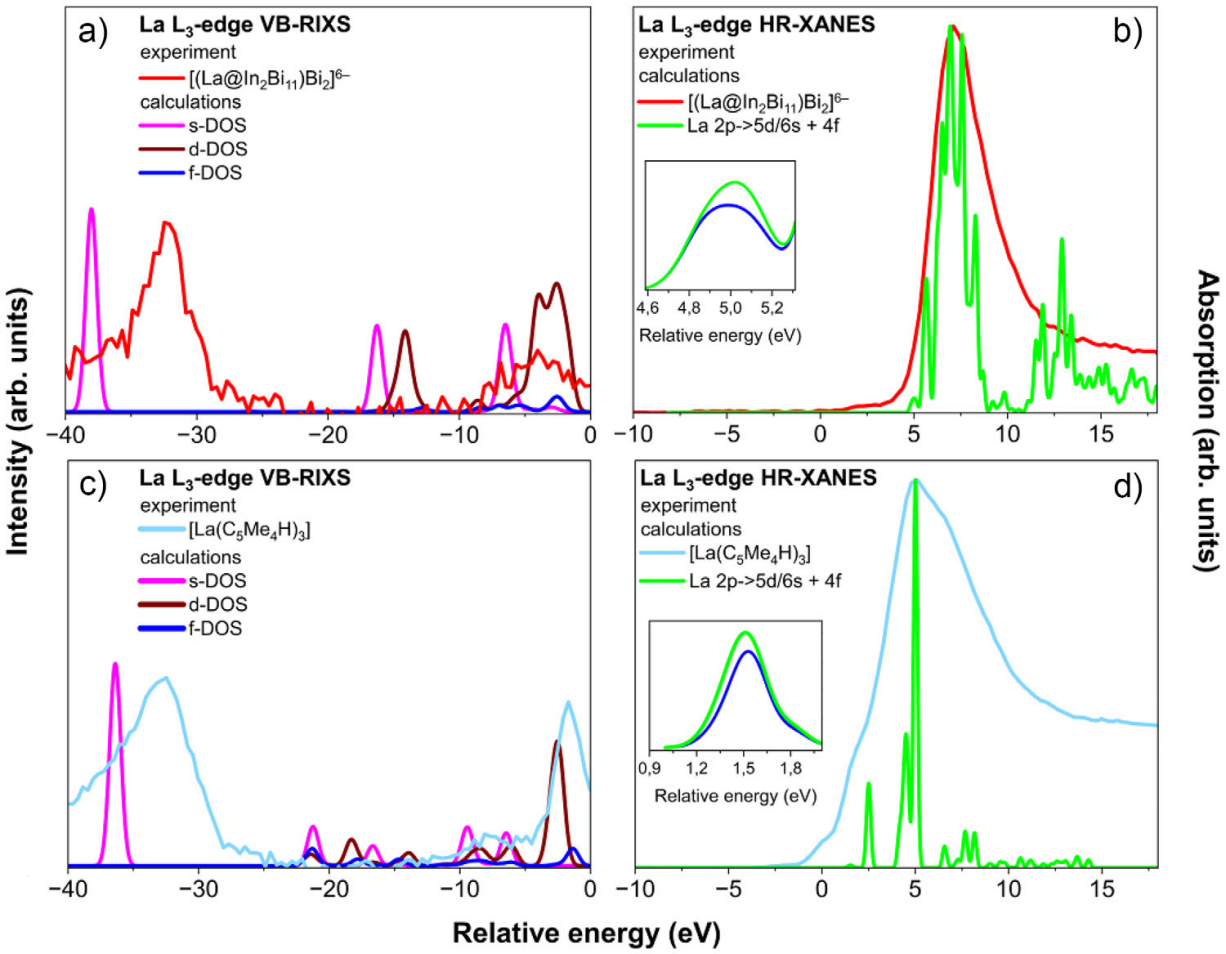
Experimental La L_3_‐edge VB‐RIXS and La L_3_‐edge HR‐XANES spectra for a), b) [K(crypt‐222)]^+^ salt of [(La@In_2_Bi_11_)_2_Bi_2_]^6−^ and c), d) for [La(C_5_Me_4_H)_3_], in comparison with the results of DFT and BSE calculations. La s, d, and f contributions to the DOS of the cluster [(La@In_2_Bi_11_)_2_Bi_2_]^6−^ (a) and the precursor [La(C_5_Me_4_H)_3_] (c). Plotted is the natural orbital population versus the quasiparticle energy as obtained from a one‐component (a) or two‐component (c) fsCD‐evGW@PBE0/x2c‐TZVPPall‐2c computations. Shown are Gaussian line shapes with full width at half maximum of 1.00 eV. Simulated La L_3_‐edge XANES spectra of the model compound “[La@In_2_Bi_11_]^4−^” (b) and [La(C_5_Me_4_H)_3_] (d), as obtained from a two‐component evGW‐BSE@PBE0/x2c‐TZVPPall‐2c computation. Shown are Gaussian line shapes with a full width at half maximum of 0.25 eV. The pre‐edge region of the spectra obtained from electric dipole transition moments are shown as solid blue lines in the insets in (b) and (d). Those obtained from both dipole and quadrupole transition moments are shown as green lines. The calculated and experimental data are aligned relative to the Fermi energy at 0 eV, and the calculated intensities in (a) and (b) are scaled for better comparison.

For directly rationalizing the experimental findings, the HR‐XANES spectra were computed and analyzed using DFT calculations followed by *GW* and BSE (*GW*‐BSE) calculations.^[^ ^19^, ^32^, ^33^ ^]^ The *GW*‐BSE approach describes the main absorption peak and (pre‐)edge region of the HR‐XANES spectra accurately (cf. Supporting Information for details on the calculations). Figure 3 displays calculated DOS and La L_3_‐edge HR‐XANES as well as experimental La L_3_‐edge HR‐XANES and VB‐RIXS spectra. The La L_3_‐edge HR‐XANES BSE calculations agree well with the experimental data (cf. Figure 3b,d).

The calculated pre‐edge features, magnified in the insets in Figure 3b,d, are shifted to higher energies compared to the experimental values, suggesting that the screening of the La 2p core‐hole, originating from the excitation process, might need to be better taken into account in the computations, which do not account for the final state 3d core‐hole, either. The pre‐edge feature is at the energy position of the La 4f unoccupied states. Due to mixing with La 5d states, it becomes better visible.

**Figure 4:**
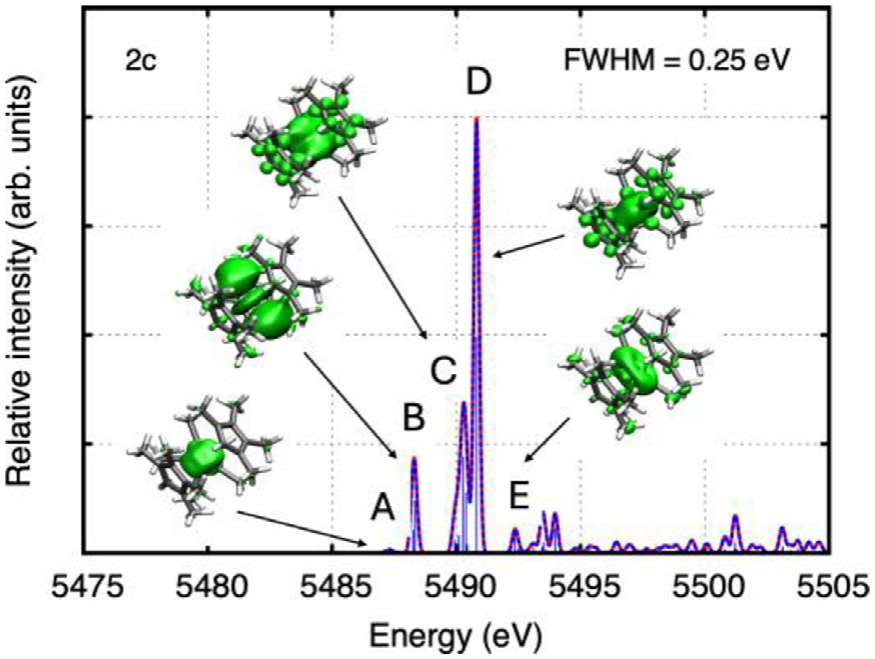
Simulated La L_3_‐edge XANES of the precursor [La(C_5_Me_4_H)_3_] as obtained from two‐component ev*GW*‐BSE@PBE0/x2c‐TZVPPall‐2c computations. Also shown are the unrelaxed particle densities for the peaks A through E. The unrelaxed particle densities are visualized at an isovalue of 0.00075 electrons/bohr.

**Figure 5:**
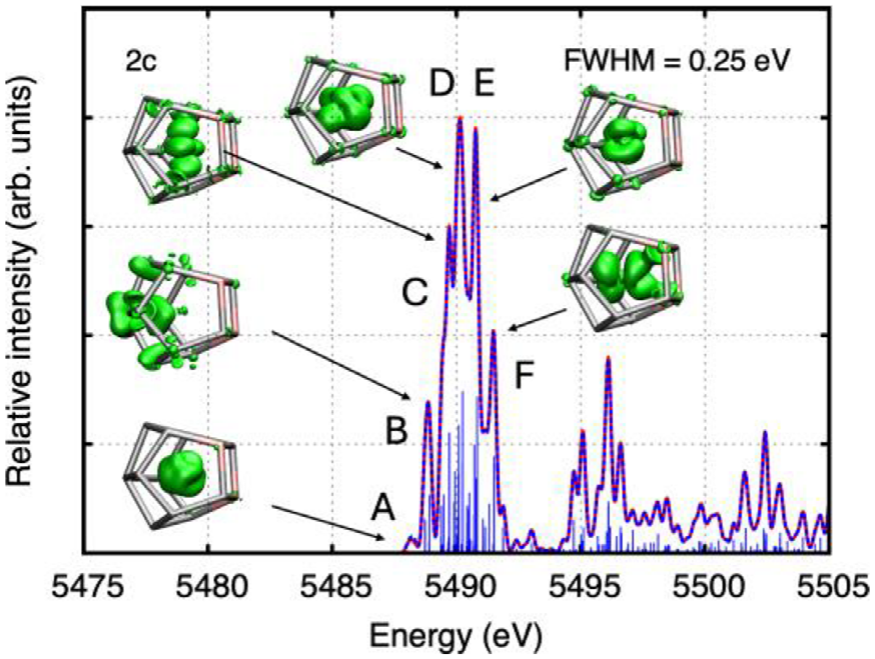
Simulated La L_3_‐edge XANES spectra of the model compound “[La@In_2_Bi_11_]^4−^” as obtained from two‐component ev*GW*‐BSE@PBE0/x2c‐TZVPPall‐2c computations. Also shown are the unrelaxed particle densities for the peaks A through F. The unrelaxed particle densities are visualized at an isovalue of 0.00075 electrons/bohr.

**Figure 6:**
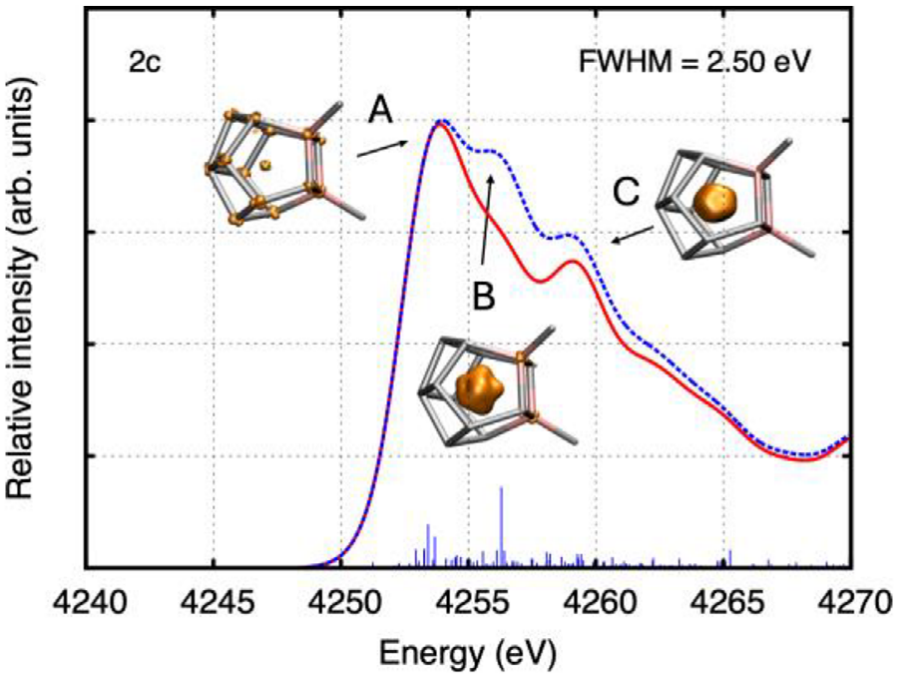
Simulated In L_1_‐edge XANES spectra of the model compound “[(La@In_2_Bi_11_)Bi_2_]^2−^” as obtained from two‐component ev*GW*‐BSE@PBE0/x2c‐TZVPPall‐2c computations. Also shown are the unrelaxed particle densities for peaks A through C. The unrelaxed particle densities are visualized at an isovalue of 0.00075 electrons/bohr^3^. Gaussian line shapes with a full width at half maximum of 2.50 eV are shown. Spectra obtained from electric dipole transition moments in the velocity representation are shown as solid red line. Those obtained from second‐order transition moments are shown as dashed blue line. For peaks A to C, the intervals [4245,4255], [4255,42581], and [42581,4262] eV were integrated, respectively.

For the visualization of specific peaks in the HR‐XANES spectra, the unrelaxed difference densities between the excited state and ground state were computed for all the excitations that contribute to a peak, weighted with the corresponding second‐order oscillator strengths.^[^ ^34^, ^35^ ^]^ The peaks were identified on the basis of a simulation with a full width at half maximum of 0.25 eV. The results are shown for [La(C_5_Me_4_H)_3_] and the closed‐shell model compounds “[La@In_2_Bi_11_]^4−^” in Figures 4 and 5 (La L_3_‐edge) and “[(La@In_2_Bi_11_)Bi_2_]^2−^” in Figure 6 (In L_1_‐edge). Note that only the unrelaxed particle densities are shown, which describe the normalized electron distribution after the core excitation of the electron. Not surprisingly, the unrelaxed hole densities are solely built from the four La 2p_3/2_ spinors (La L_3_ edge) or from the four In 2s_1/2_ spinors of the two In atoms (In L_1_‐edge) (not shown).^[^ ^36^ ^]^

**Figure 7:**
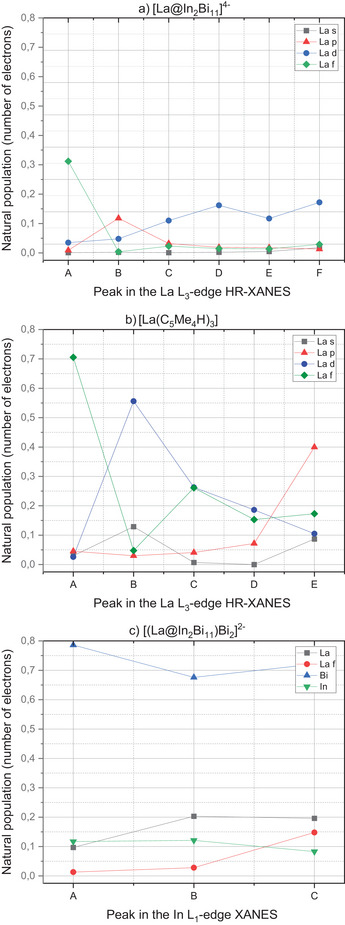
Natural populations of La s, p, d, and f shells of the unrelaxed electron densities corresponding to peaks A–D of the calculated La L_3_−edge HR‐XANES spectra for [La(C_5_Me_4_H)_3_] a) and [La@In_2_Bi_11_]^4−^ b) (cf. Figures 4 and 5), obtained from two‐component ev*GW*‐BSE@PBE0/x2c‐TZVPPall‐2c computations. c) Natural populations of La, Bi, and In from the unrelaxed electron densities corresponding to peaks A–C of the calculated In L_1_‐edge XANES spectrum of [(La@In_2_Bi_11_)Bi_2_]^2^−, obtained from two‐component ev*GW*‐BSE@PBE0/x2c‐TZVPPall‐2c computations (cf. Figure 6). Note that the peak notations (from A to E) refer to each individual spectrum; there is no relationship between the peaks in the three spectra.

Figure 7a,b show the natural population analyses of the unrelaxed particle densities of the La L_3_‐edge XANES spectra (cf. Table S3). For both [La(C_5_Me_4_H)_3_] and “[La@In_2_Bi_11_]^4−^”, the pre‐edge peak A in Figures 4 and 5 is dominated by excitations into the La 4f shell, whereas peaks B–F are dominated by excitations into the La 6s and 5d shells. Generally, La d, f, and s states appear strongly mixed with In and Bi states (cf. Figures 7c and S20c); therefore, the HR‐XANES spectra are sensitive to changes in bonding. Note that the sum of the La, Bi, and In populations is equal to one, which is the value to which the unrelaxed particle densities are normalized.

It is well known that the energy position of the rising absorption edge in the La L_3_‐edge HR‐XANES spectrum is sensitive to the electron density on the metal atom. A larger shift to lower excitation energies correlates with increased electron density on the La atom. In [La(C_5_Me_4_H)_3_], the energy shift between the pre‐edge and the rising absorption edge is barely resolved, whereas it is clearly visible for the cluster. Notably, the pre‐edges appear at similar positions in both spectra. This suggests that higher electron density is present on La in the reagent molecule, indicating greater bond covalency in the La–ligand bond compared to the La in the cluster [(La@In_2_Bi_1__1_)_2_Bi_2_]^6–^.

### La L_3_‐Edge VB‐RIXS

The La s and La d projected s‐, d‐DOS, calculated for the compounds in the ground state, describe very well the features of the VB‐RIXS spectra measured for [La(C_5_Me_4_H)_3_] and the [(La@In_2_Bi_11_)_2_Bi_2_]^6−^ anion (cf. Figure 3a,c). In a previous study, the origin of the peaks in VB‐RIXS spectra of lanthanum oxide and lanthanum carbonate was generally assigned, but no quantitative analysis or discussion of the nature of the bonds from La to neighboring atoms probed by VB‐RIXS was conducted.^[^ ^30^ ^]^ The presence of La 5d and La 6s electron density in the valence band (−10 to 0 eV) displays La−In/Bi covalency. The peaks have a larger intensity (area) for [La(C_5_Me_4_H)_3_] (0.31) as compared to [(La@In_2_Bi_11_)_2_Bi_2_]^6−^ (0.18) and La_2_O_3_ (0.11), indicating larger bond covalency with participation of La s and La d valence orbitals, similar to the HR‐XANES results. The large peaks close to −30 eV originate from core 5s→2p electron transitions. Quantitative analyses were performed using the XES NEO program, as detailed in the Supporting Information; this includes Figures S25 and S26 as well as Tables S9 and S10, where also the negligible uncertainties from the spectral modeling are listed.^[^ ^37^, ^38^ ^]^

### La–Ligand Bonding Interactions Studied by DFT

We investigated the bonding interactions of the La atom with DFT and *GW* calculations. The La atomic orbital contributions to the DOS are computed from a natural population analysis.^[^ ^39^ ^]^ The DOS peaks shown in Figures S3 through S5 are normalized such that the maximum of the highest peak is found at the value of 1.0, but when we integrate the raw (unnormalized) values, we obtain the natural populations. These are shown in Table S2. For example, when integrating the La atomic‐orbital populations over the interval [−50, −30] eV (cf. Figure 3a–c), we obtain the double occupancy of the 5s level. In the case of [(La@In_2_Bi_11_)_2_Bi_2_]^6−^, the natural population is 4.0, because there are two La centers in the full cluster. Except for this factor of two, the calculated two‐component (2c) results for “[La@In_2_Bi_11_]^4−^” and [(La@In_2_Bi_11_)_2_Bi_2_]^6−^ are very similar, indicating that the cluster “[La@In_2_Bi_11_]^4−^” is a reasonably good model system, and confirming the description of the entire cluster as a supramolecular μ‐Bi‐bridged assembly of two {La@In_2_Bi_11_} cluster units.^[^ ^40^, ^41^ ^]^ Note that there is much more charge transfer (or covalency) into the empty 5d shell than into the empty 4f shell. For example, for the model system “[La@In_2_Bi_11_]^4−^” at the 2c level, the values are 5.02 and 0.48 electrons for the 5d and 4f shells, respectively (for, 5d: 4.72, 4f: 0.45). In contrast, for the precursor molecule [La(C_5_Me_4_H)_3_], the 5d contribution is lower, whereas the 4f is higher (for 2c, 5d: 2.71, 4f: 0.71; a similar La 5d:4f ratio was found in a natural population analysis of [LaCp_3_] (Cp: cyclopentadienide).^[^ ^42^ ^]^ This result agrees with the experiments: in both compounds, there is a well distinguishable participation of 5d orbitals in covalent bonding. The experimental results suggest that the effect is larger for the precursor molecule compared to the cluster. However, it should be noted that the VB‐RIXS is recorded under resonant conditions and in the presence of a 2p core‐hole, which can lead to deviations from the ground‐state electronic structure. Moreover, minor structural changes of the cluster upon X‐ray irradiation during the VB‐RIXS experiments were present (see Supporting Information), which could have resulted in reduced La 5d participation in bonding.

**Figure 8:**
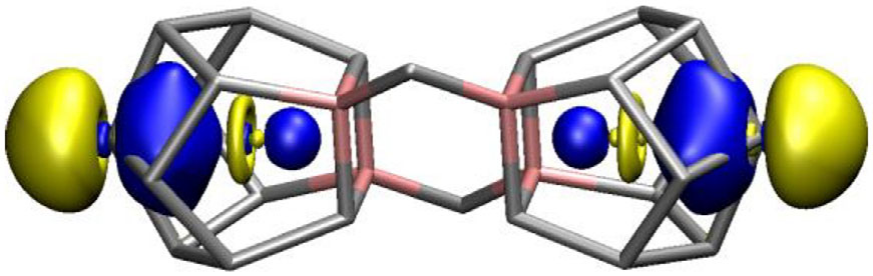
Two Pipek‐Mezey LMOs (one orbital in the left and one in the right cage) of the [(La@In_2_Bi_11_)_2_Bi_2_]^6−^ cluster showing La 5d–Bi 6p covalency through a La 5d population of 0.42 in the natural population analysis. At the PBE0/x2c‐TZVPPall‐1c level, the La s, p, d, and f populations in each of the orbitals are 0.03, 0.11, 0.42, and 0.01 electrons, respectively. The orbitals are visualized at an isovalue of 0.025 bohr^−3/2^.

When localizing the one‐component DFT molecular orbitals of the [(La@In_2_Bi_11_)_2_Bi_2_]^6−^ cluster using the Pipek‐Mezey^[^ ^43^ ^]^ approach, two doubly occupied localized molecular orbitals (LMOs) are found that show significant La d population (0.42 out of 2.00 electrons), that is, distinguishable La 5d–Bi 6p covalency. The two LMOs are depicted in Figure 8.

These LMOs can be regarded as textbook examples of the energetic stabilization of doubly occupied non‐bonding orbitals through empty atomic orbitals. Here, the empty atomic orbital is the La $5d_{z^{2}}$ orbital that stabilizes the 6p_z_ orbital of the apical Bi atom (with these atoms being situated on the *z*‐axis). The situation is similar to the well‐known examples of rare‐gas halogenides, such as XeF_2_, where an empty Xe 5d_xz_ (5d_yz_) orbital stabilizes the doubly occupied non‐bonding linear combination of the two 2p_x_ (2p_y_) orbitals of the F atoms (with these atoms being situated on the z axis). In the XeF_2_ case, at the same level of theory as applied in the present work, the Xe 5d population is only 0.07, whereas the La 5d population is 0.42 in each of the LMOs shown in Figure 8.

There are many more LMOs with noticeable 5d populations (0.05–0.10) both in the [(La@In_2_Bi_11_)_2_Bi_2_]^6−^ cluster and in the precursor, but the two LMOs shown in Figure 8 are unique. In the precursor, nine LMOs in total show La 5d populations of about 0.11–0.12 electrons (cf. Figure S12) while in the [(La@In_2_Bi_11_)_2_Bi_2_]^6−^ cluster, a total of 28 LMOs display La 5d populations in the range from 0.05 to 0.09 electrons (cf. Figures S13–S18).

### Bi L_3_‐Edge and in L_1_‐Edge XANES

**Figure 9:**
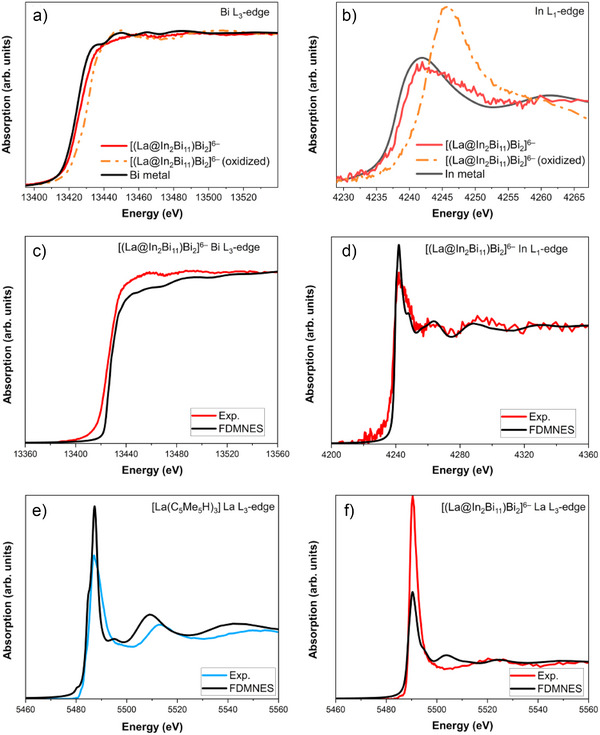
a) Bi L_3_‐edge and b) In L_1_‐edge XANES spectra of the [(La@In_2_Bi_11_)_2_Bi_2_]^6−^ cluster and the cluster exposed to air as well as Bi metal a) and In metal b). Comparison between the calculated with the FDMNES code and experimental Bi L_3_‐edge c) and In L_3_‐edge d) XANES spectra of the [(La@In_2_Bi_11_)_2_Bi_2_]^6−^ cluster. Comparison between the calculated with the FDMNES code La L_3_‐edge XANES and experimental La L_3_‐edge HR‐XANES spectra of [La(C_5_Me_4_H)_3_] precursor e) and [(La@In_2_Bi_11_)_2_Bi_2_]^6−^ cluster f). All spectra are normalized to the post‐edge region.

The large intensity and well‐defined WL, separated from the post‐edge region in the La L_3_‐edge HR‐XANES spectrum, reveal that the La electronic structure in [(La@In_2_Bi_11_)_2_Bi_2_]^6−^ is more characteristic of an atom in a positive oxidation state than of a metal atom (cf. Figure 2b). Note that the WL of a La L_3_‐edge XANES spectrum of metallic La is not that well pronounced.^[^ ^44^ ^]^ Our experiments showed that the WL intensity increases and shifts to higher energy, leading to larger pre‐edge‐WL energy shift, upon X‐ray irradiation for more than 10 minutes (cf. Figure S28). In contrast, the Bi L_3_‐edge XANES spectrum of Bi (2p → d/s) in [(La@In_2_Bi_11_)_2_Bi_2_]^6−^ is characteristic of a metallic compound, as suggested by the featureless WL with low intensity shown in Figure 9a. In addition, the spectrum is considerably shifted to lower energies compared to the spectrum of a cluster exposed to air revealing high electronic density on Bi in [(La@In_2_Bi_11_)_2_Bi_2_]^6−^. The shape of the spectrum and the energy position are very similar to the spectrum of metallic Bi (cf. Bi L_1_‐edge XANES in Figure S23). Parallel conclusions can be drawn by examining the In L_1_‐edge XANES spectrum (2s → p electron transitions) of [(La@In_2_Bi_11_)_2_Bi_2_]^6−^. The rising absorption edge is much closer to the absorption edge of the spectrum of the metallic In foil compared to the spectrum of the cluster exposed to air. There is a well‐distinguishable small energy shift to higher energies in the spectrum of the In site in the Bi‐based cluster as compared to the In foil spectrum. This illustrates that electron density from the In atom is transferred to the Bi/La atoms, and that the In atom no longer behaves as In^2−^ within the cluster environment. For the In L_1_‐edge of the model compound [(La@In_2_Bi_11_)Bi_2_]^2‐^, Figures 6 and 7c show to which atoms the core excitations from the 2s_1/2_ spinors take place (cf. Table S4). Clearly, the In L_1_‐edge excitations are dominated by excitations into Bi‐based states where In and La states are admixed. This suggests that the spectral features are primarily sensitive to changes of the In–Bi bonding interactions, but peaks B and C are also sensitive to changes of the In–La bonding. The calculated In L_1_‐edge spectrum using the *GW*‐BSE approach, depicted in Figure 6, has a spectral shape similar to the experiment (cf. Figure 9b). The second order transitions shape the WL of the spectrum.

### Local Atomic Environment Probed by XANES/HR‐XANES and FDMNES Calculations

We calculated the La L_3_‐edge XANES of [La(C_5_Me_4_H)_3_] and [(La@In_2_Bi_11_)_2_Bi_2_]^6−^, as well as the In L_1_‐edge and Bi L_3_‐edge XANES spectra of [(La@In_2_Bi_11_)_2_Bi_2_]^6−^, using the FDMNES code based on multiple scattering theory (see the Supporting Information for computational details). The calculated and experimental spectra exhibit similar shapes, namely in the post‐edge regions for all spectra, as shown in Figure 9c–f. In Figures S19–S22, the calculated DOS by FDMNES confirms that the main transition process is similar to that computed by the DFT calculations. Note that in the La L_3_‐edge XANES of [(La@In_2_Bi_11_)_2_Bi_2_]^6−^, an additional absorption peak appears in theory at 5506 eV, which is not resolved in the experiment, see Figure 9f (Figure S20a,b). The DOS reveals that this peak originates from high‐energy unoccupied orbitals of La d‐type (Figure S20b). It is likely that, due to variations of the La position in the cluster, the peak is not experimentally visible. Notably, in the La L_3_‐edge HR‐XANES spectrum of the cluster compound recorded for a sample cooled to 27 K, a small feature is visible at this energy position in the spectrum (Figure S20d), which supports the explanation given for the reduced spectral intensity at higher temperatures.

Additional FDMNES calculations were performed to investigate how dynamical atomic disorder affects the spectral shape of the La L^3^‐edge HR‐XANES spectrum (see Figure S24). We employed a normal mode sampling approach, based on vibrational calculations, to account for dynamical disorder in the La L^3^‐edge X‐ray absorption spectra of the [(La@In_2_Bi_11_)_2_Bi_2_]^6–^ cluster. In total, 84 normal modes were computed, and their frequencies are listed in Table S8. Modes primarily involving the two La atoms—through stretching, bending, or torsional motions with neighboring atoms—were selected for sampling, as they contribute significantly to structural fluctuations. For each mode, five geometries were generated by displacing atomic positions along the normal mode coordinates at ±*x*, ±0.5*x*, and 0*x*, where *x* represents the estimated displacement amplitude at the temperature used in the X‐ray experiment. We observed that the impact of disorder on the main absorption edge peak is characterized by an overall decrease in intensity, without the emergence of additional spectral features. Notably, the calculated peak located at +10 eV above the main absorption maximum—seen in FDMNES simulations without structural disorder—vanishes when vibrational modes involving torsional displacement are selectively sampled. This suggests that torsional dynamics play a critical role in modulating the spectral shape and may explain the absence of this peak in the experimental data (cf. Figure S24). The FDMNES results give evidence that the experimental structures of the [(La@In_2_Bi_11_)_2_Bi_2_]^6−^ cluster and the [La(C_5_Me_4_H)_3_] precursor obtained from X‐ray diffraction used in the calculations provide a reasonable representation of the experimental samples studied when dynamical disorder is considered.

## Conclusion

We investigated and compared the bonding properties of La in the [(La@In_2_Bi_11_)_2_Bi_2_]^6−^ cluster, and the [La(C_5_Me_4_H)_3_] reactant used for its synthesis, by La L_3_‐edge HR‐XANES and VB‐RIXS experimental techniques, along with DFT and *GW*‐BSE calculations performed with TURBOMOLE and FDMNES. Both experiments and calculations demonstrated that La incorporated into the Bi‐based cage exhibits clearly distinguishable covalent interaction mainly with the Bi from the cage. The calculations suggest that La 5d participation in covalent bonding with ligands is stronger in the cluster with a substantial La 5d orbital participation in one specific molecular orbital, reminiscent of the binding interaction described for XeF_2_. The experiments confirm stronger La–ligand bond covalency in the precursor as compared to the cluster. However, it should be noted that the spectroscopic tools probe the excited state of the La atom, so the results may, to some extent, differ from ground‐state calculations. It was shown that the electronic structure of the La atoms in [(La@In_2_Bi_11_)_2_Bi_2_]^6−^ is more characteristic of a molecule, in which the interactions of the Bi and In atoms exhibit a predominantly metallic character. Electron charge transfer from In to Bi/La is likely, as previously proposed by calculations.

This insight into the stabilization of intermetalloid clusters by covalent contributions involving the interstitial atom was achieved by pushing the limits of state‐of‐the‐art experimental and computational techniques. We consider this an important result, as an ongoing debate concerns the extent to which lanthanide atoms form covalent bonds via their 4f and/or 5d valence orbitals. Our findings reveal the presence of a symbiotic intermetallic bonding, which was demonstrated here for the first time.

We envisage that these results will inspire new synthetic strategies to enhance covalent interactions between interstitial lanthanide atoms and a cluster shell in which they are embedded, potentially facilitating new elemental combinations and leading to novel cluster architectures with unique physical and chemical properties. Similarly, precursors with even more variable covalent interactions between Ln atoms and (in)organic ligands will be tested for the synthesis of intermetalloid clusters, in order to explore their influence on the respective formation mechanisms.

The experimental and theoretical methods we used are highly versatile and applicable to any lanthanide compounds; therefore, this study outlines a path forward for future investigations into binding–stability/reactivity relationships in intermetallic clusters and other lanthanide‐based species. This will also encompass studies of photochemical properties and their relation to bonding characteristics.

In summary, the spectroscopic approach presented herein, including quantitative analyses of VB‐RIXS spectra using genetic algorithms, offers an advanced toolset to deepen the understanding of bonding in multimetallic clusters, and as such helps to further the research in this area.

## Supporting Information

The authors have cited additional references within the Supporting Information.^[^ ^45^, ^46^, ^47^, ^48^, ^49^, ^50^, ^51^, ^52^, ^53^, ^54^, ^55^, ^56^, ^57^, ^58^, ^59^, ^60^, ^61^, ^62^, ^63^, ^64^, ^65^, ^66^, ^67^, ^68^, ^69^, ^70^, ^71^, ^72^, ^73^, ^74^, ^75^ ^]^ The Supporting Information is available free of charge at: https://onlinelibrary.wiley.com/action/downloadSupplement?doi=10.1002%2Fanie.202512019&file=anie202512019‐s1‐SuppInf_202512019.pdf. The Supporting Information contains: details on DFT based calculations (DFT and *GW*‐BSE) with TURBOMOLE, details on multiple scattering‐based calculations with the FDMNES code, comparison of experimental and computed spectra, and experimental details.

## Conflict of Interests

The authors declare no conflict of interest.

## Supporting information



Supporting Information

## Data Availability

The data that support the findings of this study are available from the corresponding author upon reasonable request.
