# Supplementary material for: Molecular‐Metallic Binding Characteristics of the Intermetalloid f‐/p‐Block Cluster [(La@In2Bi11)2Bi2]6−
Source: Angew Chem Int Ed Engl. 2025 Aug 4;64(37):e202512019. doi: 10.1002/anie.202512019 (PMC12416455; doi:10.1002/anie.202512019)
Supplement: Supplementary file 1 — Supporting Information [file ANIE-64-e202512019-s001.pdf]

Supplementary Information

Molecular-Metallic Binding Characteristics of the Intermetalloid f-/p-Block Cluster $[(La@In_2Bi_{11})_2Bi_2]^{6-}$

Harry Ramanantoanina,[a] Julia Rienmüller,[b] Yannick R. Lohse,[b] Nina Rauwolf,[c] Max Kehry,[c] Cedric Reitz,[a] Emily Marie Reynolds,[a] Tim Prüßmann,[a] Bianca Schacherl,[a] Viktoriia A. Saveleva,[d] Ruwini S. K. Ekanayake,[a] Jörg Göttlicher,[e] Bastian Weinert,[b] Wim Klopper,[b,c]* Stefanie Dehnen,[b]* Tonya Vitova[a]*

[a] Dr. H. Ramanantoanina, C. Reitz, E. M. Reynolds, Dr. T. Prüßmann, Dr. B. Schacherl, Dr. R. S. K. Ekanayake, Prof. Dr. T. Vitova
Institute for Nuclear Waste Disposal
Karlsruhe Institute of Technology
Kaiserstr. 12, 76131 Karlsruhe, Germany
E-mail: tonya.vitova@kit.edu

[b] Dr. J. Rienmüller, Y.R. Lohse, Dr. B. Weinert, Prof. Dr. W. Klopper, Prof. Dr. S. Dehnen
Institute of Nanotechnology
Karlsruhe Institute of Technology
Kaiserstr. 12, 76131 Karlsruhe, Germany
E-mail: stefanie.dehnen@kit.edu, willem.klopper@kit.edu

[c] N. Rauwolf, Dr. M. Kehry, Prof. Dr. W. Klopper
Institute of Physical Chemistry
Karlsruhe Institute of Technology
Kaiserstr. 12, 76131 Karlsruhe, Germany
E-mail: willem.klopper@kit.edu

[d] Dr. V.A. Saveleva
European Synchrotron Radiation Facility (ESRF)
71, avenue des Martyrs, CS 40220, 38043 Grenoble Cedex 9, France

[e] Dr. J. Göttlicher
Institute for Photon Science and Synchrotron Radiation
Karlsruhe Institute of Technology

Contents

1 Quantum-chemical Computations 2
1.1 Density of States 3
1.2 X-Ray Absorption Spectra 6
1.3 Equilibrium Geometries 12
2 FDMNES calculations 13
3 Experimental details 20
4 References 27

1 Quantum-chemical Computations

Quantum-chemical computations were performed with the TURBOMOLE program package, version 7.8.[1,2] The COSMO model [3] was applied in all computations with default parameters (note that the option “r all b” had to be used in the radius-definition menu of the cosmoprep script of TURBOMOLE; for recent work on the COSMO model, not exploited here, see Ref. [4]).

The equilibrium structures of the precursor $[La(C_5Me_4H)_3]$ and of the cluster $[(La@In_2Bi_{11})_2Bi_2]^{6-}$ were optimized at the density-functional-theory (DFT) level using the PBE0 functional [5,6] and the x2c-TZVP-Pall basis set [7] of atomic Gaussian functions. The PBE0 functional was chosen in view of the subsequent *GW* computations (*G* is the Green’s function and *W* is the screened electron-electron interaction) and in view of the computations in the framework of the Bethe-Salpeter equation (BSE). It is our standard functional that has proven to perform well on the GW100 test set of ionization energies [8,9] as well as with respect to the computation of excitation energies at the Bethe-Salpeter level.[10] The resolution-of-the-identity (RI) approximation was used when constructing the matrix representation of the Coulomb operator, for which the x2c-TZVPPall auxiliary basis set [7] was used (“jbas” in TURBOMOLE jargon). The geometry optimizations were performed at the one-component (1c) level accounting for scalar-relativistic effects by means of including the 1c part of the one-electron exact-two-component (X2C) Hamiltonian.[11] A finite-nucleus model was used [12] and the diagonal, local approximation to the unitary decoupling transformation (DLU) was applied.[12] With respect to convergence of the equilibrium structure, the convergence criterion for the energy was 10^{-6} E_h and the convergence criterion for the maximum norm of the DFT gradient was 10^{-3} E_h/a_0 (default parameters). With respect to convergence of the Kohn–Sham equations, the convergence criterion for the DFT energy was 10^{-9} E_h. For numerical integration, TURBOMOLE’s grid 4a was used throughout the present work, which has been designed for all-electron quasi-relativistic computations.[13]

The optimized structures of the precursor $[La(C_5Me_4H)_3]$ and of the cluster $[(La@In_2Bi_{11})_2Bi_2]^{6-}$ are shown in Figures S1 and S2. The corresponding Cartesian coordinates are given in Tables S5 and S6. The Cartesian coordinates of the model systems $[La@In_2Bi_{11}]^{4-}$ and $[(La@In_2Bi_{11})Bi_2]^{2-}$ are subsets of the coordinates of the full cluster $[(La@In_2Bi_{11})_2Bi_2]^{6-}$. The equilibrium structures of $[La(C_5Me_4H)_3]$ and $[(La@In_2Bi_{11})_2Bi_2]^{6-}$ display C_3 and D_{2h} point-group symmetry, respectively, but all computations of the present work were performed without exploiting symmetry, that is, they were performed in C_1 symmetry.

At the optimized structures, single-point computations were performed at the *GW* level to obtain one-electron quasiparticle energies, and single-point BSE computations were performed to obtain core-excitation energies to simulate the La L_3- and In L_1-edge x-ray absorption spectra. The *GW* computations are described in Section 1.1 and the BSE computations in Section 1.2.

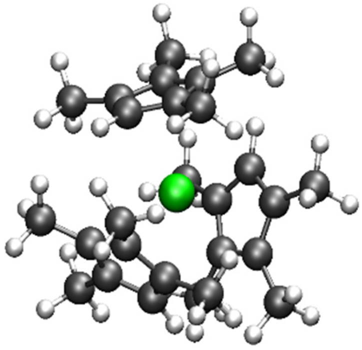

Figure S1. Structure of the optimized, C_3-symmetric structure of $[La(C_5Me_4H)_3]$. Green: La; black: C; white: H.

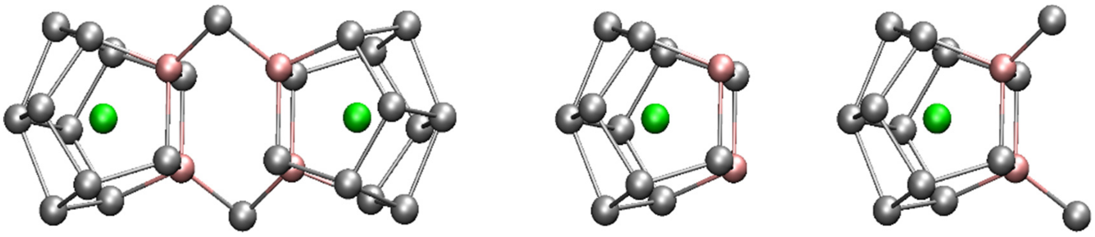

Figure S2. Structures of the systems studied computationally. From left to right: $[(La@In_2Bi_{11})_2Bi_2]^{6-}$, $[La@In_2Bi_{11}]^{4-}$, and $[(La@In_2Bi_{11})Bi_2]^{2-}$. The Cartesian coordinates of the latter two structures are kept fixed to those in the parent system $[(La@In_2Bi_{11})_2Bi_2]^{6-}$. Green: La; gray: Bi, pink: In. The point-group symmetries are D_{2h}, C_{2v}, and C_{2v}, respectively.

1.1 Density of States

Quasiparticle energies were computed by means of the *GW* approach at the scalar-relativistic 1c level as described above as well as at the two-component level (2c) accounting for spin-orbit coupling. At the 2c level, the screened nuclear spin-orbit (SNSO) approach [12] was used with the parameter $snsopara set to 1, which is the default parameter for X2C computations. Furthermore, Kramers symmetry was enforced using the keyword $kramers.

We used the eigenvalue-self-consistent variant ev*GW* in conjunction with the contour-deformation technique employing frequency sampling (fsCD-ev*GW*) as implemented in the TURBOMOLE program package.[14,15] In view of the BSE computations to be performed subsequently to the ev*GW* calculations, the one-particle levels treated explicitly in the ev*GW* computations were those of the *L* shell of lanthanum in preparation of the simulation of the La L_3-edge x-ray absorption spectra, the 2s orbitals (or spinors) of In in preparation of the simulation of the In L_1-edge spectra, and a few dozen of levels about the Fermi level as documented in Table S1 (column "contours (ev*GW*)").

All *GW* and BSE computations were performed in the x2c-TZVPPall-2c basis set,[7] also the scalar-relativistic 1c computations. For the *GW* and BSE computations, an auxiliary basis set of the type "cbas" (in TURBOMOLE jargon) is required. This auxiliary basis was provided by Harding.[16]

Table S1. One- (1c) and two-component (2c) fsCD-ev*GW*-BSE@PBE0/x2c-TZVPPall-2c computations performed on $[(La@In_2Bi_{11})_2Bi_2]^{6-}$, $[La(C_5Me_4H)_3]$, and the model compounds $[La@In_2Bi_{11}]^{4-}$ and $[(La@In_2Bi_{11})Bi_2]^{2-}$. Given are the contours (orbitals respectively spinors) explicitly considered in the fsCD-ev*GW* computation, the orbitals respectively spinors kept frozen in the core-valence-separation (CVS) approach, and the number of excited states computed using the BSE method.

System	Type	Edge	Contours (ev*GW*)	Frozen orbs. (CVS)	M_S	States
$[(La@In_2Bi_{11})_2Bi_2]^{6-}$	1c	La L_3-edge	127–134, 1111–1183	135–1154	0	1200
					1	1200
		In L_1-edge	135–138, 1111–1183	139–1154	0	1200
					1	1200
	2c	La L_3-edge	253–268, 2221–2366	269–2308	–	n.a.[1)]
		In L_1-edge	269–276, 2221–2366	277–2308	–	n.a.[1)]
$[La(C_5Me_4H)_3]$	1c	La L_3-edge	2–5, 124–136	6–129	0	400
					1	400
	2c	La L_3-edge	3–10, 247–272	11–258	–	1600
$[La@In_2Bi_{11}]^{4-}$	1c	La L_3-edge	59–62, 516–547	63–536	0	800
					1	800
		In L_1-edge	63–64, 516–547	65–536	0	800
					1	800
	2c	La L_3-edge	117–124, 1031–1094	125–1072	–	1600
		In L_1-edge	125–128, 1031–1094	129–1072	–	1600
$[(La@In_2Bi_{11})Bi_2]^{2-}$	1c	La L_3-edge	69–72, 596–634	73–618	0	800
					1	800
		In L_1-edge	73–74, 596–634	75–618	0	800
					1	800
	2c	La L_3-edge	137–144, 1191–1266	145–1236	–	1600
		In L_1-edge	145–148, 1191–1266	149–1236	–	1600

1) n.a. = not available.

In Figures S3 through S5, we show densities of states (DOS) of the precursor $[La(C_5Me_4H)_3]$, the model system $[La@In_2Bi_{11}]^{4-}$, and the full cluster $[(La@In_2Bi_{11})_2Bi_2]^{6-}$ as obtained from 2c computations. The intensities of the peaks are determined by the contributions of lanthanum atomic orbitals (or spinors) to the molecular spinors of the molecule. The relative energies with respect to the Fermi level at 0 keV refer to the quasiparticle energies obtained from the fsCD-ev*GW*@PBE0/x2c-TZVPPall-2c computations. These quasiparticle energies are negative for occupied and positive for unoccupied spinors.

Not shown is the lanthanum p DOS, which is of less interest. Note that the fully occupied 5p shell of La occurs in the range of energies from −0.03 to −0.02 keV. At about −0.04 keV, the doubly occupied 5s shell of La is found. Of most interest are the d and f densities of states of La(III), that is, the contributions from the empty 5d and 4f shells of La(III) to molecular orbitals. This may also be understood as the amount of charge transfer from the cluster cage onto the La^{3+} ion.

Two-component (2c) fsCD-ev*GW*@PBE0/x2c-TZVPPall-2c computations were performed to compare the model compound $[La@In_2Bi_{11}]^{4-}$ with the full cluster $[(La@In_2Bi_{11})_2Bi_2]^{6-}$. In the model compound, the La $2p_{3/2}$ level was found at −5.49213 keV while this level was found at −5.49231 keV in the full cluster. The small difference of only 0.18 eV indicates that the model compound is well suited to model the full cluster. The peak maximum of the La f-DOS of the model compound was found at 0.00746 keV (Figure S4) while the peak maximum of the La f-DOS of the full cluster was found at 0.00701 keV (Figure S5). Here the difference amounts to 0.45 eV, and the effect on the 4f–2p energy gap is only 0.27 eV (less than 0.1 ‰).

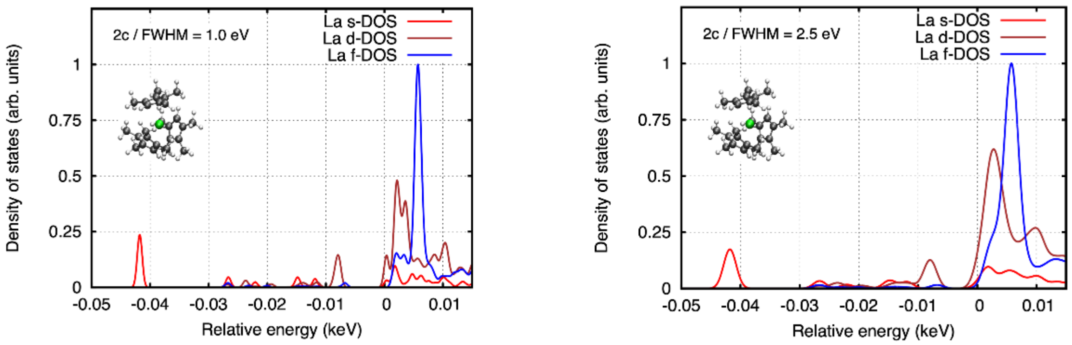

Figure S3. Lanthanum s-, d-, and f-contributions to the density of states (DOS) of the precursor [La(C_5Me_4H)$_3$]. Plotted is the natural orbital population versus the quasiparticle energy as obtained from a two-component fsCD-ev*GW*@PBE0/x2c-TZVPPall-2c computation. Shown are Gaussian line shapes with full width at half maximum of 1.00 and 2.50 eV.

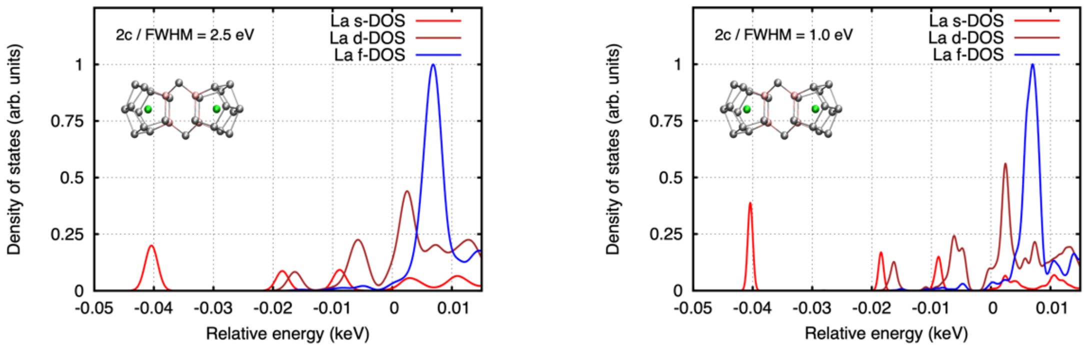

Figure S4. Lanthanum s-, d-, and f-contributions to the density of states (DOS) of [La@In_2Bi_{11}]$^{4-}$. Plotted is the natural orbital population versus the quasiparticle energy as obtained from a two-component fsCD-ev*GW*@PBE0/x2c-TZVPPall-2c computation. Shown are Gaussian line shapes with full width at half maximum of 1.00 and 2.50 eV.

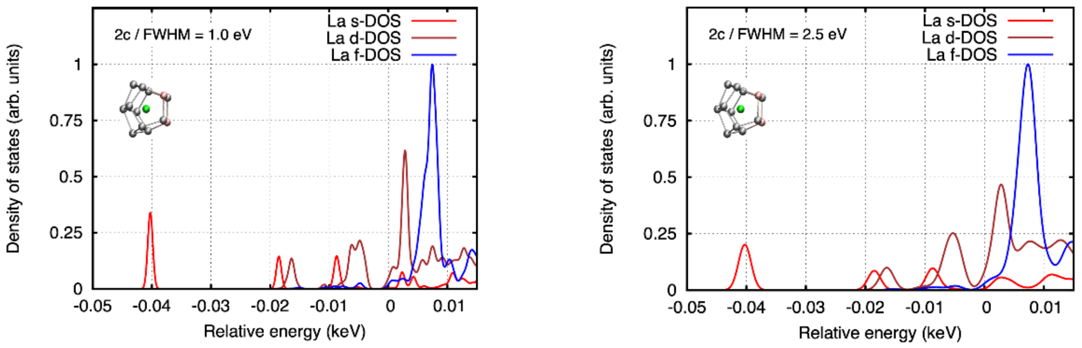

Figure S5. Lanthanum s-, d-, and f-contributions to the density of states (DOS) of [(La@In_2Bi_{11})$_2$$Bi_2$]$^{6-}$. Plotted is the natural orbital population versus the quasiparticle energy as obtained from a two-component fsCD-ev*GW*@PBE0/x2c-TZVPPall-2c computation. Shown are Gaussian line shapes with full width at half maximum of 1.00 and 2.50 eV.

Table S2. Natural populations of lanthanum s-, p-, d- and f-shells as obtained by integrating the density of states of Figures S3–S5 (2c results) on the intervals [–0.05,–0.03] and [–0.03,0.0] keV.

			Natural population (number of electrons)			
**System**	**Type**	**Interval (keV)**	**La s**	**La p**	**La d**	**La f**
[La(C_5Me_4H)$_3$]	1c	[–0.05,–0.03]	2.00	0.00	0.00	0.00
		[–0.03,0.0]	1.44	7.88	2.61	0.76
	2c	[–0.05,–0.03]	2.00	0.00	0.00	0.00
		[–0.03,0.0]	1.38	8.43	2.71	0.71
[La@In_2Bi_{11}]$^{4-}$	1c	[–0.05,–0.03]	2.00	0.00	0.00	0.00
		[–0.03,0.0]	2.16	9.68	4.72	0.45
	2c	[–0.05,–0.03]	2.00	0.00	0.00	0.00
		[–0.03,0.0]	1.95	9.60	5.02	0.48
[(La@In_2Bi_{11})$_2Bi_2$]$^{6-}$	1c	[–0.05,–0.03]	4.00	0.00	0.00	0.00
		[–0.03,0.0]	4.29	19.17	9.44	1.21
	2c	[–0.05,–0.03]	4.00	0.00	0.00	0.00
		[–0.03,0.0]	3.80	19.13	9.44	1.13

1.2 X-Ray Absorption Spectra

Core-excitation energies were computed by means of the Bethe-Salpeter equation (BSE) as implemented in the TURBOMOLE program package by Krause and Klopper at the 1c level [17] and by Holzer and Klopper at the 2c level.[14] All core-excitation energies were computed at the ev*GW*-BSE@PBE0/x2c-TZVPPall-2c level. The core-valence-separation (CVS) approach [18,19] was employed, meaning that the occupied spinors (or orbitals) between the relevant core level and the Fermi level were kept frozen, that is, omitted from the computation. The relevant core level is the La $2p_{3/2}$ level in case of the simulation of the La L_3-edge x-ray absorption spectra and the In $2s_{1/2}$ level in case of the simulation of the In L_1-edge spectra.

We have computed oscillator strengths in the dipole approximation, that is, from electric dipole transition moments in the velocity approximation (zeroth-order oscillator strengths), as well as second-order oscillator strengths obtained by adding electric-quadrupole, electric-octupole, magnetic-dipole, and magnetic-quadrupole contributions to the zeroth-order oscillator strengths, following the work by Bernadotte *et al.*[20] The total second-order oscillator strengths calculated in this manner are origin independent and the approach was implemented by Kehry in the TURBOMOLE program package.[21]

In Figures S6 through S11, the spectra obtained from the zeroth-order oscillator strengths are shown as solid red curves while those obtained from the second-order oscillator strengths are shown as dashed blue curves. The difference between the zeroth- and second-order oscillator strengths is highlighted in Figure S11, which displays the pre-edge features of the La L_3-pre-edge x-ray absorption spectra of the precursor [La(C_5Me_4H)$_3$] and of the model compound [La@In_2Bi_{11}]$^{4-}$.

Table S3. Natural populations of lanthanum s-, p-, d- and f-shells of the unrelaxed particle densities obtained from two-component ev*GW*-BSE@PBE0/x2c-TZVPPall-2c computations.

			Natural population (number of electrons)			
**System**	**Edge**	**Peak**	**La s**	**La p**	**La d**	**La f**
[La(C_5Me_4H)$_3$]	La L_3	A	0.030	0.007	0.026	0.307
		B	0.129	0.000	0.556	0.008
		C	0.007	0.005	0.263	0.105
		D	0.000	0.019	0.186	0.056
		E	0.087	0.168	0.105	0.065
[La@In_2Bi_{11}]$^{4-}$	La L_3	A	0.001	0.009	0.035	0.312
		B	0.002	0.118	0.048	0.004
		C	0.001	0.032	0.110	0.023
		D	0.002	0.019	0.162	0.015
		E	0.005	0.018	0.117	0.014
		F	0.018	0.013	0.172	0.029

Table S4. Natural populations of La, Bi, and In of the unrelaxed particle densities obtained from two-component ev*GW*-BSE@PBE0/x2c-TZVPPall-2c computations. The La f population is given in parenthesis.

			Natural population (number of electrons)		
**System**	**Edge**	**Peak**	**La (f)**	**Bi**	**In**
[(La@In_2Bi_{11})Bi_2]$^{2-}$	In L_1	A	0.097 (0.013)	0.786	0.117
		B	0.203 (0.028)	0.676	0.121
		C	0.196 (0.148)	0.721	0.083

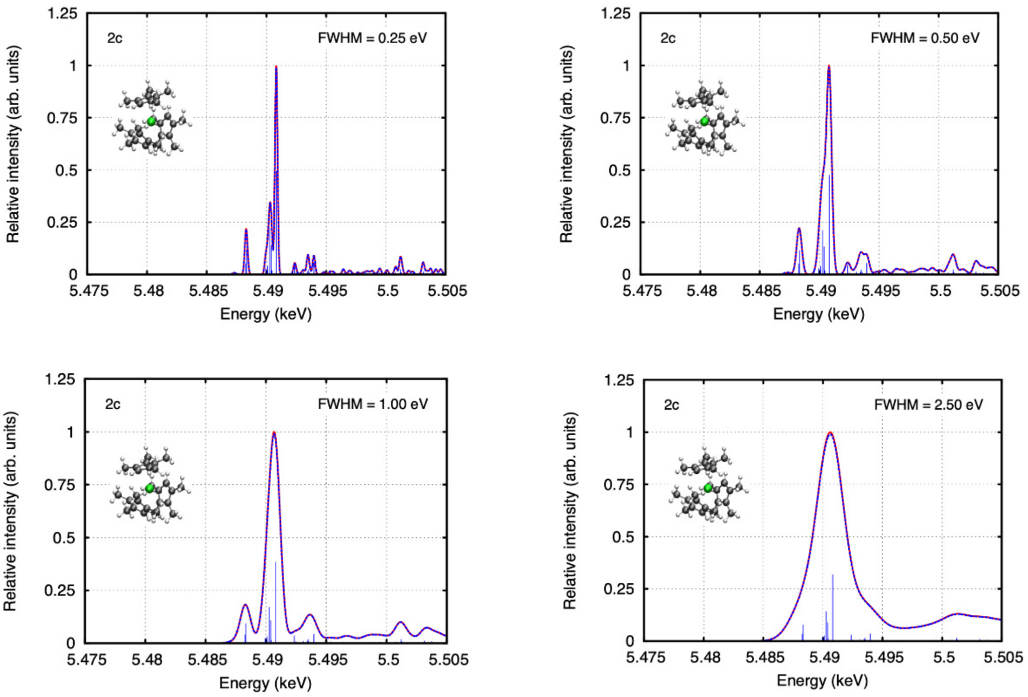

Figure S6. Simulated La L_3-edge x-ray absorption spectra of the precursor [La(C_5Me_4H)$_3$] as obtained from a two-component ev*GW*-BSE@PBE0/x2c-TZVPPall-2c computation. Shown are Gaussian line shapes with full width at half maximum of 0.25, 0.50, 1.00, and 2.50 eV. Spectra obtained from electric dipole transition moments in the velocity representation are shown as solid red line. Those obtained from second-order transition moments are shown as dashed blue line.

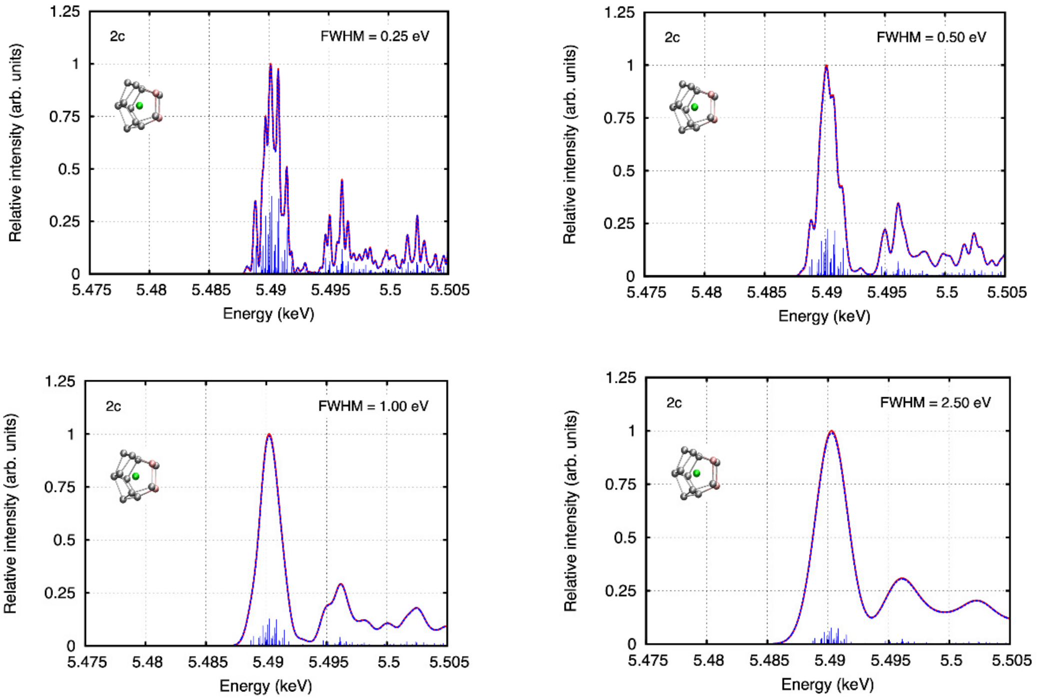

Figure S7. Simulated La L_3-edge x-ray absorption spectra of the model compound $[La@In_2Bi_{11}]^{4-}$ as obtained from a two-component ev*GW*-BSE@PBE0/x2c-TZVPPall-2c computation. Shown are Gaussian line shapes with full width at half maximum of 0.25, 0.50, 1.00, and 2.50 eV. Spectra obtained from electric dipole transition moments in the velocity representation are shown as solid red line. Those obtained from second-order transition moments are shown as dashed blue line.

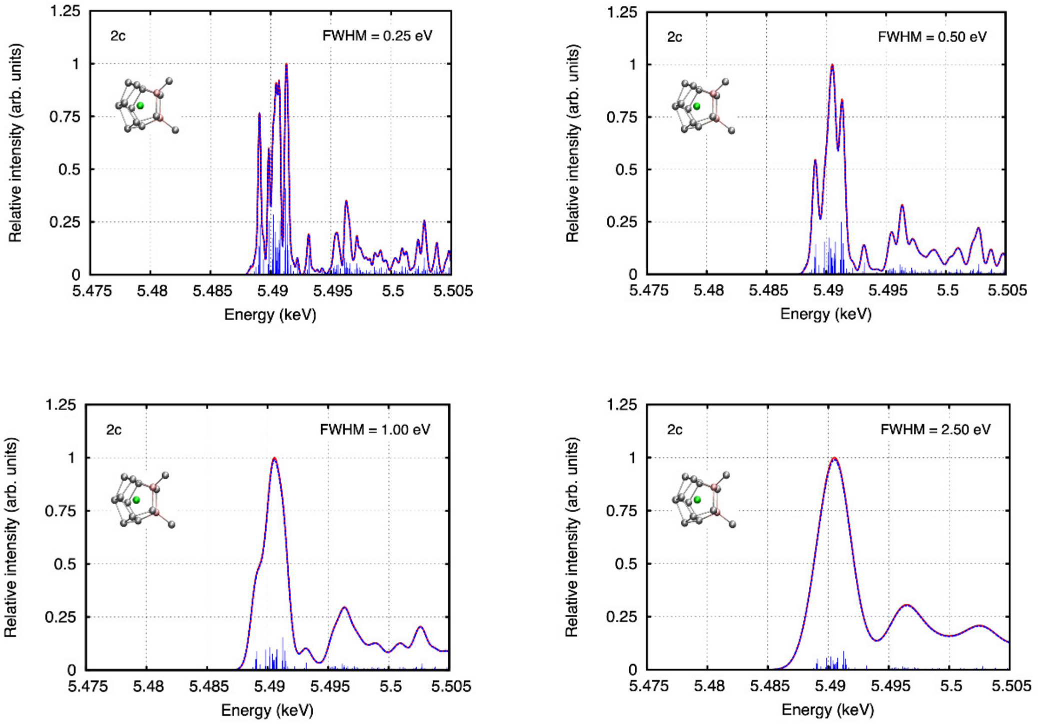

Figure S8. Simulated La L_3-edge x-ray absorption spectra of the model compound $[(La@In_2Bi_{11})Bi_2]^{2-}$ as obtained from a two-component ev*GW*-BSE@PBE0/x2c-TZVPPall-2c computation. Shown are Gaussian line shapes with full width at half maximum of 0.25, 0.50, 1.00, and 2.50 eV. Spectra obtained from electric dipole transition moments in the velocity representation are shown as solid red line. Those obtained from second-order transition moments are shown as dashed blue line.

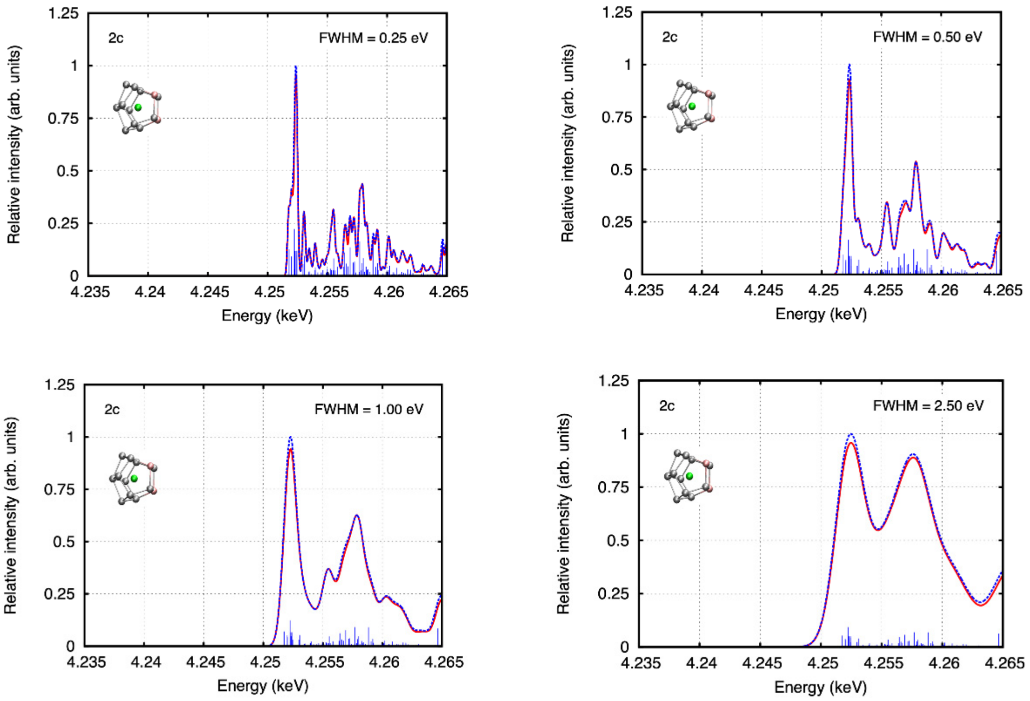

Figure S9. Simulated In L_1-edge x-ray absorption spectra of the model compound $[La@In_2Bi_{11}]^{4-}$ as obtained from a two-component ev*GW*-BSE@PBE0/x2c-TZVPPall-2c computation. Shown are Gaussian line shapes with full width at half maximum of 0.25, 0.50, 1.00, and 2.50 eV. Spectra obtained from electric dipole transition moments in the velocity representation are shown as solid red line. Those obtained from second-order transition moments are shown as dashed blue line.

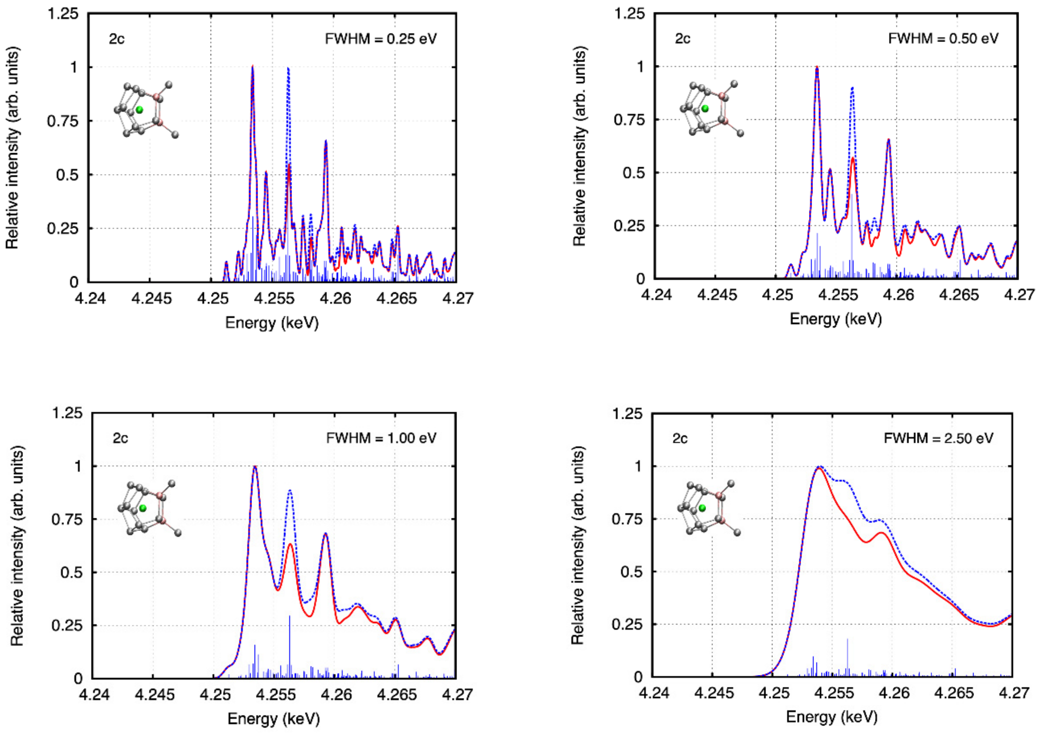

Figure S10. Simulated In L_1-edge x-ray absorption spectra of the model compound $[(La@In_2Bi_{11})Bi_2]^{2-}$ as obtained from a two-component ev*GW*-BSE@PBE0/x2c-TZVPPall-2c computation. Shown are Gaussian line shapes with full width at half maximum of 0.25, 0.50, 1.00, and 2.50 eV. Spectra obtained from electric dipole transition moments in the velocity representation are shown as solid red line. Those obtained from second-order transition moments are shown as dashed blue line.

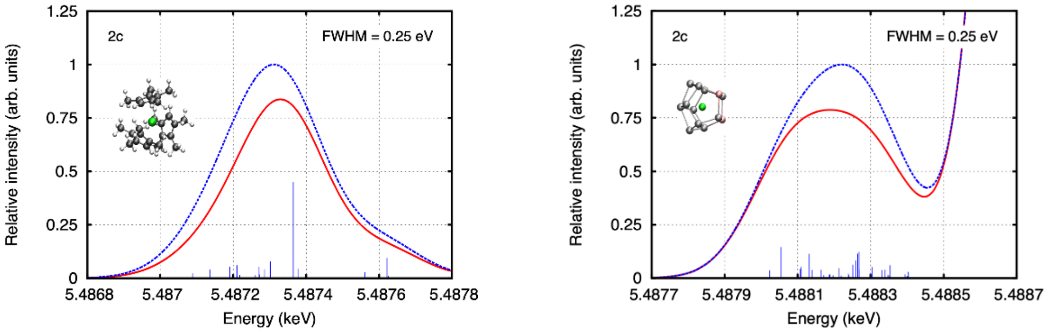

Figure S11. Simulated La L_3-pre-edge x-ray absorption spectra of the precursor [La(C_5Me_4H)$_3$] (left) and of the model compound [La@In_2Bi_{11}]$^{4-}$ (right) as obtained from two-component ev*GW*-BSE@PBE0/x2c-TZVPPall-2c computations. Shown are Gaussian line shapes with full width at half maximum of 0.25 eV. Spectra obtained from electric dipole transition moments in the velocity representation are shown as solid red line. Those obtained from second-order transition moments are shown as dashed blue line.

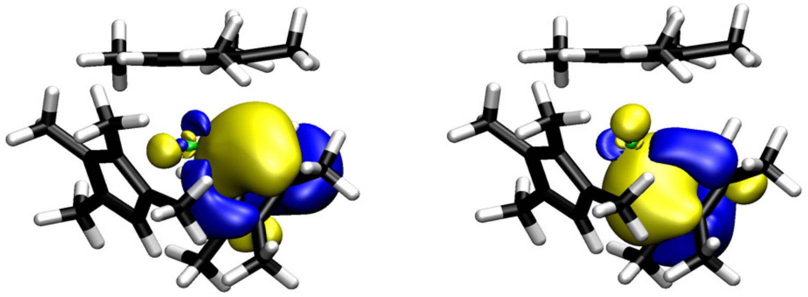

Figure S12. Localized molecular orbitals (LMOs) as obtained from applying the Pipek–Mezey approach to the precursor [La(C_5Me_4H)$_3$] at the one-component PBE0/x2c-TZVPPall-2c level. Left: one out of six equivalent LMOs with La s, p, d, and f populations of 0.00, 0.02, 0.12, and 0.03 electrons, respectively. Right: one out of three equivalent LMOs with La s, p, d, and f populations of 0.02, 0.03, 0.11, and 0.04 electrons, respectively. The orbitals are visualized at an isovalue of 0.025 bohr$^{-3/2}$.

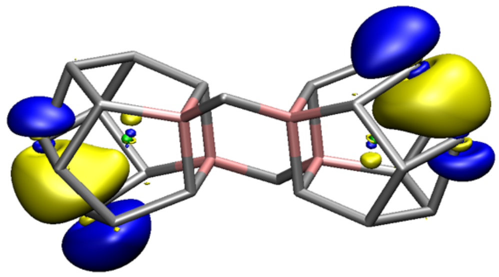

Figure S13. Two localized molecular orbitals (LMOs) as obtained from applying the Pipek–Mezey approach to the full cluster [(La@In_2Bi_{11})$_2$$Bi_2$]$^{6-}$ at the one-component PBE0/x2c-TZVPPall-2c level. The two equivalent LMOs have La s, p, d, and f populations of 0.00, 0.01, 0.09, and 0.02 electrons, respectively. The orbitals are visualized at an isovalue of 0.025 bohr$^{-3/2}$.

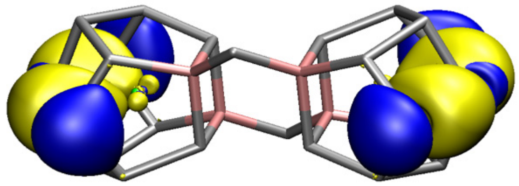

Figure S14. Four localized molecular orbitals (LMOs) as obtained from applying the Pipek–Mezey approach to the full cluster [(La@In_2Bi_{11})$_2$$Bi_2$]$^{6-}$ at the one-component PBE0/x2c-TZVPPall-2c level. The four equivalent LMOs have La s, p, d, and f populations of 0.00, 0.01, 0.09, and 0.02 electrons, respectively. The orbitals are visualized at an isovalue of 0.025 bohr$^{-3/2}$.

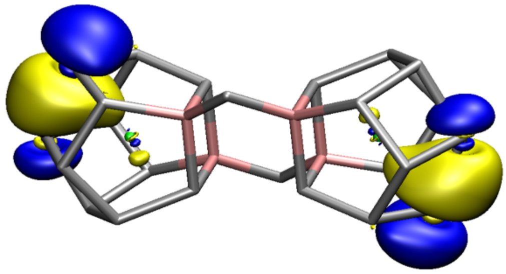

Figure S15. Two localized molecular orbitals (LMOs) as obtained from applying the Pipek–Mezey approach to the full cluster $[(La@In_2Bi_{11})_2Bi_2]^{6-}$ at the one-component PBE0/x2c-TZVPPall-2c level. The two equivalent LMOs have La s, p, d, and f populations of 0.00, 0.01, 0.09, and 0.02 electrons, respectively. The orbitals are visualized at an isovalue of 0.025 $bohr^{-3/2}$.

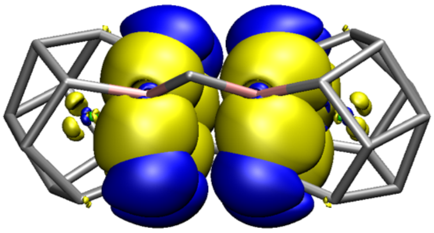

Figure S16. Eight localized molecular orbitals (LMOs) as obtained from applying the Pipek–Mezey approach to the full cluster $[(La@In_2Bi_{11})_2Bi_2]^{6-}$ at the one-component PBE0/x2c-TZVPPall-2c level. The eight equivalent LMOs have La s, p, d, and f populations of 0.00, 0.01, 0.09, and 0.02 electrons, respectively. The orbitals are visualized at an isovalue of 0.025 $bohr^{-3/2}$.

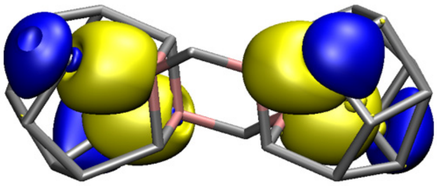

Figure S17. Four localized molecular orbitals (LMOs) as obtained from applying the Pipek–Mezey approach to the full cluster $[(La@In_2Bi_{11})_2Bi_2]^{6-}$ at the one-component PBE0/x2c-TZVPPall-2c level. The four equivalent LMOs have La s, p, d, and f populations of 0.00, 0.03, 0.08, and 0.01 electrons, respectively. The orbitals are visualized at an isovalue of 0.025 $bohr^{-3/2}$.

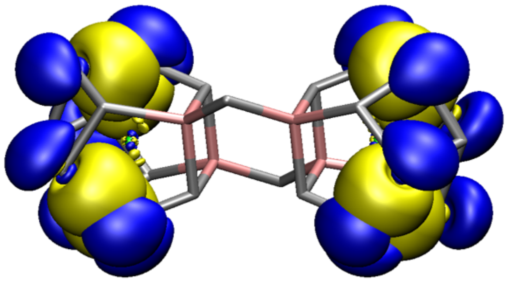

Figure S18. Eight localized molecular orbitals (LMOs) as obtained from applying the Pipek–Mezey approach to the full cluster $[(La@In_2Bi_{11})_2Bi_2]^{6-}$ at the one-component PBE0/x2c-TZVPPall-2c level. The eight equivalent LMOs have La s, p, d, and f populations of 0.00, 0.01, 0.05, and 0.01 electrons, respectively. The orbitals are visualized at an isovalue of 0.025 $bohr^{-3/2}$.

1.3 Equilibrium Geometries

Table S5. Cartesian coordinates (in Å) of the precursor [La(C_5Me_4H)$_3$] as optimized at the PBE0/x2c-TZVPPall level in the present work.

67

PBE0/x2c-TZVPPall + COSMO + 1c-X2C optimization (charge=0)

La	0.0000733	0.0000110	0.0190451
C	0.2092672	2.7716188	0.0195374
C	-0.5717180	2.5195180	-1.1298244
C	-1.8636357	2.1365102	-0.6888475
H	1.2369571	3.1041055	0.0199686
C	-0.1687883	2.7430840	-2.5546274
H	-0.3359114	1.8674577	-3.1912376
H	0.8861770	3.0063572	-2.6272538
H	-0.7421249	3.5601018	-3.0047485
C	-3.0573702	1.9845748	-1.5740466
H	-2.7872321	1.6069359	-2.5619546
H	-3.5517055	2.9506771	-1.7299225
H	-3.8070168	1.3123253	-1.1537070
C	-2.5052566	-1.2039563	0.0195382
C	-1.8966456	-1.7544321	-1.1298609
C	2.2956593	-1.5672875	0.0194085
C	2.4680482	-0.7648822	-1.1299364
C	-0.5725503	2.5189786	1.1682355
C	-1.8642095	2.1363004	0.7261587
C	-1.8956616	-1.7548247	1.1682426
H	-3.3068164	-0.4799297	0.0200083
C	-0.9185189	-2.6821859	0.7261350
C	2.4679675	-0.7640274	1.1681711
H	2.0697020	-2.6235305	0.0197836
C	2.7825272	0.5458942	0.7261432
C	-3.0587485	1.9845770	1.6103273
H	-3.8076729	1.3114496	1.1900970
H	-2.7893720	1.6081742	2.5989134
H	-3.5537904	2.9505655	1.7645733
C	-0.9190514	-2.6818383	-0.6888901
C	-2.2921912	-1.5176248	-2.5545891
H	-1.4506279	-1.2246221	-3.1916116
H	-3.0477820	-0.7357408	-2.6271267
H	-2.7132360	-2.4227884	-3.0043009
C	-0.1906333	-3.6398629	-1.5739468
H	0.0009053	-3.2175478	-2.5621191
H	-0.7799127	-4.5512802	-1.7291578
H	0.7666129	-3.9524836	-1.1537748
C	2.7824711	0.5453739	-0.6888660
C	2.4610333	-1.2256093	-2.5547525
H	1.7858150	-0.6438972	-3.1916010
H	2.1627714	-2.2712091	-2.6274706
H	3.4553129	-1.1365115	-3.0045255
C	3.2479985	1.6552074	-1.5738803
H	2.7863365	1.6100907	-2.5619865
H	4.3319163	1.6004110	-1.7292317
H	3.0403067	2.6405243	-1.1536339
C	3.2484382	1.6562659	1.6102949
H	2.7885251	1.6106665	2.5991960
H	4.3326708	1.6025519	1.7637743
H	3.0390715	2.6414176	1.1904799
C	2.4602961	-1.2236653	2.5933563
H	1.7847958	-0.6414054	3.2294131
H	2.1619304	-2.2692144	2.6666834
H	3.4543494	-1.1342867	3.0435877
C	-0.1896881	-3.6408637	1.6101575
H	0.0010888	-3.2197974	2.5989994
H	-0.7782960	-4.5529796	1.7637496
H	0.7680374	-3.9521262	1.1900727
C	-2.2897384	-1.5180723	2.5934073
H	-1.4472581	-1.2262628	3.2297594
H	-3.0443374	-0.7352897	2.6668026
H	-2.7115017	-2.4228283	3.0432739
C	-0.1705028	2.7417856	2.5934184
H	-0.3399440	1.8664506	3.2297919
H	0.8849306	3.0029639	2.6669671
H	-0.7425460	3.5599379	3.0431365

Table S6. Cartesian coordinates (in Å) of the cluster [(La@In_2Bi_{11})$_2$$Bi_2$]$^{6-}$ as optimized at the PBE0/x2c-TZVPPall level in the present work.

30

PBE0/x2c-TZVPPall + COSMO + 1c-X2C optimization (charge=-6)

Bi	12.1239568	0.8396088	21.5562969
Bi	12.2511883	4.7909269	19.5549191
Bi	9.6473677	1.2713219	17.3401658
Bi	11.4132389	2.5881940	15.3399088
Bi	12.8516271	-1.4681900	19.8332636
Bi	16.2605090	1.9853041	19.1758871
Bi	11.2842279	-1.2099266	17.2650537
Bi	15.4204548	-0.7613655	18.4100508
Bi	15.5397522	3.0313613	16.4848609
Bi	15.5668546	4.1285016	23.3398565
Bi	13.0515613	4.7317541	16.6947316
Bi	13.7935992	0.3333951	15.7464411
La	12.9182155	1.6818714	18.3635269
In	14.2003107	3.0356041	21.1187364
In	10.1720524	2.5991052	20.0008947
Bi	11.1155461	6.5436057	22.9371129
Bi	10.9941525	2.5927421	24.9400758
Bi	13.5951474	6.1172672	27.1514425
Bi	11.8323757	4.7992578	29.1536213
Bi	10.3852057	8.8511950	24.6592189
Bi	6.9816477	5.3932104	25.3206501
Bi	11.9547003	8.5962322	27.2264487
Bi	7.8184096	8.1414502	26.0847484
Bi	7.7056985	4.3490374	28.0114831
Bi	7.6755058	3.2553189	21.1538928
Bi	10.1963065	2.6524728	27.8008908
Bi	9.4487810	7.0505310	28.7474045
La	10.3240201	5.7016094	26.1304649
In	9.0418827	4.3452173	23.3764595
In	13.0702842	4.7866261	24.4922215

2 FDMNES calculations

The calculation of the La L_3-edge, In L_1-edge and Bi L_1-edge XANES and HR-XANES spectra of the $La(C_5Me_4H)_3$ precursor and $[(La@In_2Bi_{11})Bi_2]^{6-}$ cluster were obtained with the FDMNES code [22] using the finite difference method.[23] The electronic structure was calculated using DFT with a local exchange-correlation potential.[22] Relativistic effects were included in the calculation using the Dirac-Slater approach, and spin-orbit coupling was taken into consideration for the core-electrons. Electric-quadrupole transitions were also included in the simulation. The calculated spectra were broadened using a *pseudo*-Voigt function, which is weighted sum of a Lorentzian and a Gaussian function. The Lorentzian function simulates the core-hole lifetime broadening (Γ_{HWHM} = 1.50 eV for the In L_1-edge and La L_3-edge spectra, and Γ_{HWHM} = 2.20 eV for the Bi L_3-edge spectra), whereas the Gaussian function simulates the experimental broadening (Γ_{HWHM} = 0.25 eV). We used the default FDMNES parameter values for the step functions that mimic the absorption jump of the corresponding metal edges. Example for FDMNES input file is shown in Table S7. The calculated La L_3-edge spectrum for $La(C_5Me_4H)_3$ is shown in Figure S19. The calculated spectra for $[(La@In_2Bi_{11})Bi_2]^{6-}$ are shown in Figure S20, S21 and S22 for La L_3-edge, In L_1-edge and Bi L_3-edge, respectively.

Table S7. FDMNES input file for La L_3-edge XANES of the $La(C_5Me_4H)_3$ precursor.

```
Filout
  LaPrecursor_LaL3_mol_out

Edge
  L3

Z_absorber
  57

Range
  -80 0.1 10. 0.2 20. 1. 50. 5 150

Molecule
  1.0 1.0 1.0 90.0 90.0 90.0
  57   0.00000000   0.00000000   0.00000000
  6   -2.68388242   0.82307916   0.00000000
  6   -2.14623619   1.45096488  -1.15559131
  6   -1.29421581   2.50802647  -0.71140977
  6   -1.29421581   2.50802647   0.71140977
  6   -2.14623619   1.45096488   1.15559131
  6   -2.49832392   1.15905286  -2.59099133
  6   -0.69960385   3.56226292  -1.60121812
  6   -0.69960385   3.56226292   1.60121812
  6   -2.49832392   1.15905286   2.59099133
  6    0.62913375  -2.73584994   0.00000000
  6    2.05474868   1.91277078   0.00000000
  6   -0.18345435  -2.58417751  -1.15559131
  6    2.32969054   1.13321263  -1.15559131
  6   -1.52490673  -2.37483701  -0.71140977
  6    2.81912254  -0.13318947  -0.71140977
  6   -1.52490673  -2.37483701   0.71140977
  6    2.81912254  -0.13318947   0.71140977
  6   -0.18345435  -2.58417751   1.15559131
  6    2.32969054   1.13321263   1.15559131
  6    0.24539274  -2.74313841  -2.59099133
  6    2.25293118   1.58408555  -2.59099133
  6   -2.73520826  -2.38700616  -1.60121812
  6    3.43481211  -1.17525676  -1.60121812
  6   -2.73520826  -2.38700616   1.60121812
  6    3.43481211  -1.17525676   1.60121812
  6    0.24539274  -2.74313841   2.59099133
  6    2.25293118   1.58408555   2.59099133
  1   -3.14598844   0.28239568  -2.66532855
```

```
   1     -3.03055129     2.00458779     -3.04725658
   1     -1.61467134     0.97671869     -3.21805234
   1     -0.45059472     3.16653823     -2.59095537
   1     -1.41230604     4.38570262     -1.75621356
   1      0.20769854     4.00173997     -1.17574062
   1     -3.14598844     0.28239568      2.66532855
   1     -1.61467134     0.97671869      3.21805234
   1     -3.03055129     2.00458779      3.04725658
   1      0.20769854     4.00173997      1.17574062
   1     -1.41230604     4.38570262      1.75621356
   1     -0.45059472     3.16653823      2.59095537
   1      3.36175921    -2.18074219      1.17574062
   1      4.50428290    -0.96975840      1.75621356
   1      2.96759991    -1.19304264      2.59095537
   1      1.81755606     2.58330807      2.66532855
   1      1.65319887     0.90998705      3.21805234
   1      3.25129960     1.62224051      3.04725658
   1      2.96759991    -1.19304264     -2.59095537
   1      4.50428290    -0.96975840     -1.75621356
   1      3.36175921    -2.18074219     -1.17574062
   1      1.81755606     2.58330807     -2.66532855
   1      3.25129960     1.62224051     -3.04725658
   1      1.65319887     0.90998705     -3.21805234
   1      1.32843238    -2.86570375      2.66532855
   1     -0.03852753    -1.88670574      3.21805234
   1     -0.22074831    -3.62682831      3.04725658
   1     -3.56945774    -1.82099778      1.17574062
   1     -3.09197686    -3.41594422      1.75621356
   1     -2.51700519    -1.97349559      2.59095537
   1     -2.51700519    -1.97349559     -2.59095537
   1     -3.09197686    -3.41594422     -1.75621356
   1     -3.56945774    -1.82099778     -1.17574062
   1      1.32843238    -2.86570375     -2.66532855
   1     -0.22074831    -3.62682831     -3.04725658
   1     -0.03852753    -1.88670574     -3.21805234
   1     -3.38260614    -0.00419575      0.00000000
   1      1.69493670    -2.92732497      0.00000000
   1      1.68766944     2.93152073      0.00000000
Radius
  6.5
Delta_E_conv
  0.02
quadrupole
Density
state_all
SCF
Relativiste
Spinorbite
Convolution
Gamma_max
  15
Gamma_hole
  1.50
Ecent
  30
Elarg
  30
End
```

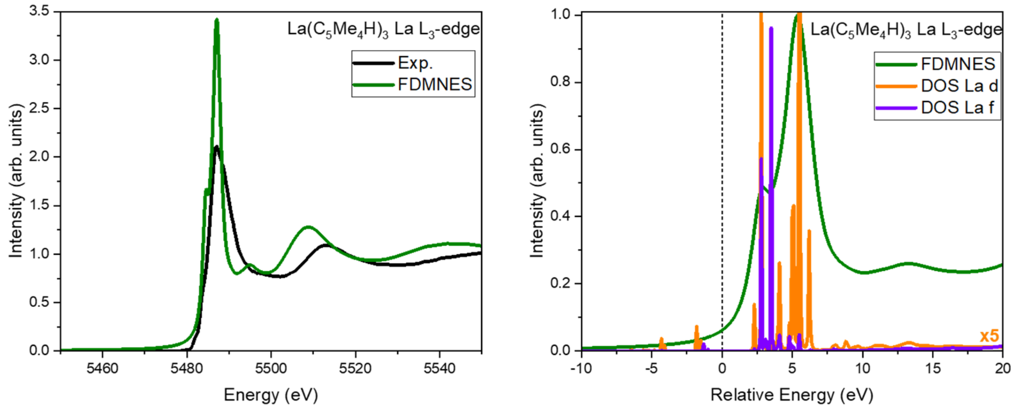

Figure S19. Comparison between the theoretical (FDMNES) and experimental (Exp.) La L_3-edge HR-XANES spectra of $La(C_5Me_4H)_3$ precursor (a), together with the projected La f and d orbitals density of state (DOS) in the vicinity of the main absorption band (white line) (b). Note that in (a) the spectra are normalized to the post-edge energy, and the theoretical excitation energy is shifted in order to align the white lines of the theoretical data with the experiment. Note also that in (b) the x-axis is given relative to the Fermi energy, allowing a better representation of the calculated DOS and the calculated La L_3-edge white line.

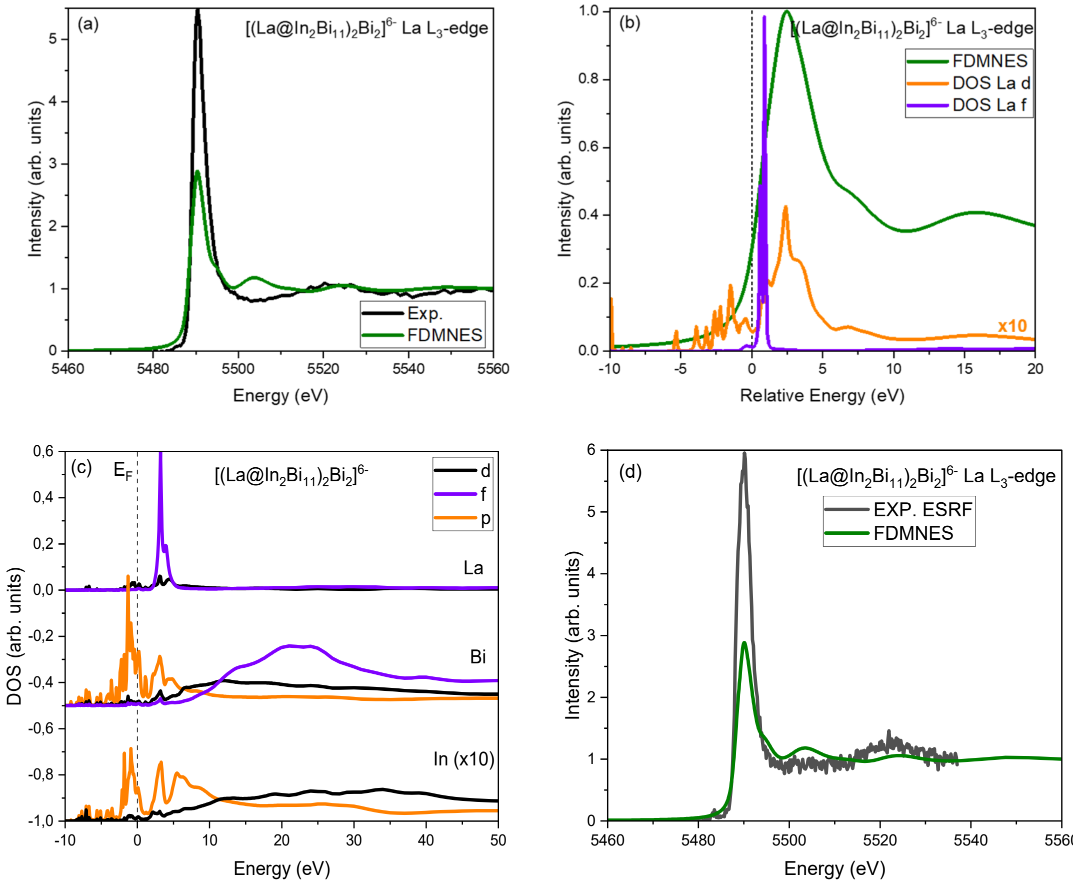

Figure S20. Comparison between the theoretical (FDMNES) and experimental (Exp.) La L_3-edge HR-XANES spectra of $[(La@In_2Bi_{11})Bi_2]^{6-}$ cluster (a), together with the projected La f and d orbitals density of state (DOS) in the vicinity of the main absorption band (white line) (b); note that to obtain the spectrum in (a) the La $L\beta_2$ characteristic fluorescence line is recorded at the KIT Light Source. Comparison of La, Bi and In p-, d- or f-DOS (c). (d) The same as (a) but the experimental spectrum is obtained by recording the La Lα characteristic fluorescence at ESRF and the sample is cooled to 27 K. Note that in (a) the spectra are normalized to the post-edge energy, and the theoretical excitation energy is shifted in order to align the white lines of the theoretical data with the experiment. Note also that in (b) the x-axis is given relative to the Fermi energy, allowing a better representation of the calculated DOS and the calculated La L_3-edge white line.

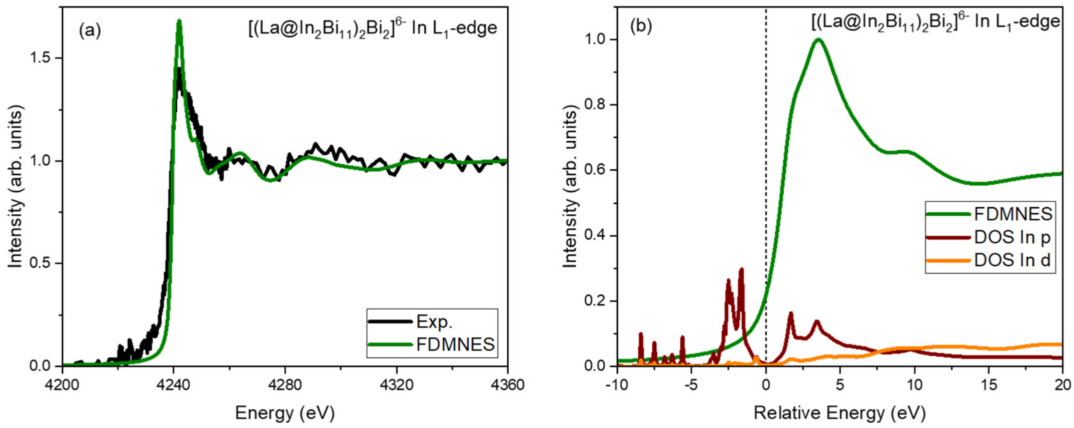

Figure S21. Comparison between the theoretical (FDMNES) and experimental (Exp.) In L_1-edge XANES spectra of $[(La@In_2Bi_{11})Bi_2]^{6-}$ cluster (a), together with the projected In p and d orbitals density of state (DOS) in the vicinity of the main absorption band (white line) (b). Note that in (a) the spectra are normalized to the post-edge energy, and the theoretical excitation energy is shifted in order to align the white lines of the theoretical data with the experiment. Note also that in (b) the x-axis is given relative to the Fermi energy, allowing a better representation of the calculated DOS and the calculated In L_1-edge white line.

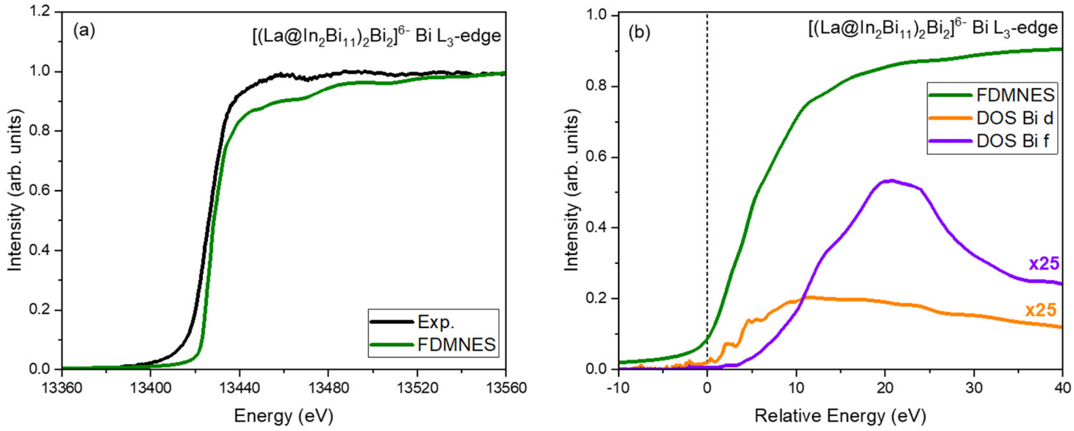

Figure S22. Comparison between the theoretical (FDMNES) and experimental (Exp.) Bi L_3-edge HR-XANES spectra of $[(La@In_2Bi_{11})Bi_2]^{6-}$ cluster (a), together with the projected In d and f orbitals density of state (DOS) in the vicinity of the main absorption band (white line) (b Note that in (a) the spectra are normalized to the post-edge energy, and the theoretical excitation energy is shifted in order to align the white lines of the theoretical data with the experiment. Note also that in (b) the x-axis is given relative to the Fermi energy, allowing a better representation of the calculated DOS and the calculated Bi L_3-edge white line.

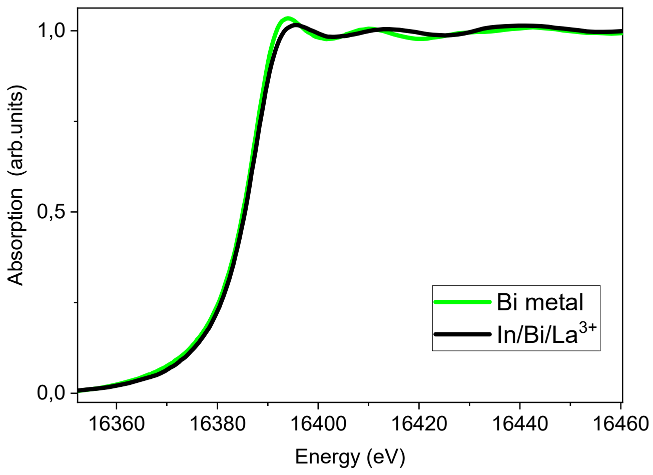

Figure S23. Bi L_1-edge XANES of Bi metal and $[(La@In_2Bi_{11})Bi_2]^{6-}$ cluster.

Disorder effects were included by employing a normal mode sampling approach based on vibrational calculations. The structure of the cluster $[(La@In_2Bi_{11})_2Bi_2]^{6-}$ was re-optimized and vibrational frequency calculations were performed using DFT (Amsterdam Density Functional; PBE functional; relativistic corrections via the zeroth order regular approximation (ZORA) Hamiltonian; Slater-type orbitals TZP basis set [24]). In total, 84 normal modes were computed, the optimized coordinates and the frequencies are listed in Table S8. Note that the structural symmetry was reduced from D_{2h} (Table S6) to C_2. This symmetry lowering enabled the convergence of the vibrational frequency analysis without imaginary modes, indicating also that the optimized C_2 structure corresponds to a local minimum on the potential energy surface within the present DFT set up.

Normal modes that primarily involve the two La atoms, in stretching, bending, or torsional motions with neighboring atoms, were selected for the sampling, as they contribute significantly to structural fluctuations. For each mode, five structures were generated by displacing atomic positions along the normal mode coordinates at ±x, ±0.5x, and 0x, where x estimates the displacement amplitude at the temperature of the experiment. The X-ray absorption spectra were computed using FDMNES for each distorted structure and are shown in Figure S24 (also in the main text, Figure 10b). The final spectrum that accounts for dynamical disorder in the La L_3-edge X-ray absorption spectra was obtained by averaging all contributions and applying Gaussian broadening to simulate thermal effects (Figure S24 and Figure 10b).

Table S8. Cartesian coordinates (in Å) and calculated vibrational frequencies (in cm^{-1}) of the cluster $[(La@In_2Bi_{11})_2Bi_2]^{6-}$ obtained from DFT PBE/ZORA-STO(TZP).

La	0.01593133733621	4.65302497444372	0.00223149706903
La	-0.01593133733621	-4.65302497444372	0.00223149706903
In	0.00341355738868	2.04850276214707	2.13338780110850
In	-0.01380321485161	-2.04798027442695	-2.12699403569391
In	0.01380321485161	2.04798027442695	-2.12699403569391
In	-0.00341355738868	-2.04850276214707	2.13338780110850
Bi	0.01039027448136	7.77124862657224	-0.01215977404652
Bi	2.18629818975122	6.43392545531267	2.18720372504847
Bi	-2.15715903536435	6.44428207134122	2.18949727510740
Bi	2.18378129420482	6.43698978635680	-2.19630226703104
Bi	-2.15319517473100	6.44287598268138	-2.18493835232339
Bi	-3.54773902407239	4.76554743754608	0.00323327803410
Bi	3.57631111508542	4.76278266444659	-0.00327873038926
Bi	0.01024983948535	4.77497838961520	-3.52528062368474
Bi	0.01313853007600	4.78351649456579	3.52561549220069
Bi	-2.23765471067051	2.02325957104959	0.00150772548993
Bi	2.26558277318453	2.02395923671784	0.00371672408380
Bi	0.00000000000000	0.00000000000000	-4.17116986312011
Bi	-0.00000000000000	-0.00000000000000	4.17629041317400
Bi	-2.26558277318453	-2.02395923671784	0.00371672408380
Bi	2.23765471067051	-2.02325957104959	0.00150772548993
Bi	-3.57631111508542	-4.76278266444659	-0.00327873038926
Bi	-0.01024983948535	-4.77497838961520	-3.52528062368474
Bi	3.54773902407239	-4.76554743754608	0.00323327803410
Bi	-0.01313853007600	-4.78351649456579	3.52561549220069
Bi	2.15715903536435	-6.44428207134122	2.18949727510740
Bi	-2.18629818975122	-6.43392545531267	2.18720372504847
Bi	2.15319517473100	-6.44287598268138	-2.18493835232339
Bi	-2.18378129420482	-6.43698978635680	-2.19630226703104
Bi	-0.01039027448136	-7.77124862657224	-0.01215977404652

Frequency (cm^{-1})	Absorption Intensity (km/mole)	irrep
7.7352	0.2053	b
6.5165	0.0004	a
9.7278	0.0269	a
12.0570	0.0106	b
15.3124	0.1089	a
16.6191	0.0019	a
20.3862	0.0057	b
24.7884	4.4720	b
30.4825	0.1298	b
32.1986	0.3242	a
32.4951	0.0295	a
32.8540	4.5083	b
33.1159	4.9237	a
33.2413	0.6461	b
34.1060	0.0097	b
36.8008	0.0096	a
37.2896	0.1658	b
38.3519	18.2235	a
43.5771	0.6645	
44.2792	0.6376	
44.8573	2.8399	a
45.5939	2.6653	b
45.7446	6.4056	
46.0160	1.4489	
47.7890	0.0189	b
51.7634	0.8093	a
51.9125	6.2852	b
52.0178	0.7456	a
52.6744	7.5012	b
55.6953	0.0114	b
56.0981	0.0687	a
56.2092	0.0031	a
57.0063	0.8258	a
58.3482	2.7294	b
58.5155	17.8025	b
60.3488	0.0240	a
64.0880	0.3953	b
64.8922	1.8351	a
68.1094	1.5285	b
68.6036	0.0488	
68.6432	0.0280	
68.7622	0.0380	a
73.6008	1.4519	b
73.7837	0.0330	a
79.9912	1.2754	b
81.6662	0.1979	a
82.2017	27.1140	b
83.4008	4.2750	a
88.3863	0.0098	b
89.7259	3.6422	
89.8481	4.9018	a
90.1750	4.5393	a
95.1607	0.0146	b
95.2744	0.0045	a
99.2813	0.0485	b
100.1514	0.0172	a
101.0421	1.1818	b
101.7857	0.0025	a
102.4626	0.1304	b
102.9842	0.7149	a
103.9903	13.9610	b
104.2421	0.3326	a
105.9514	0.0354	b
106.0606	0.0013	a
109.5308	0.6102	a
109.8246	3.3656	b
110.7176	12.4056	b
111.2981	0.0256	a
111.8480	0.1041	b
113.2799	0.0130	a
114.8844	0.0777	b
116.8920	9.5901	a
117.9339	2.2891	b
119.7522	0.0564	a
124.8701	0.0452	b
127.4899	17.2910	a
128.0532	0.7913	b
128.4334	1.0606	a
140.0056	243.8611	b
145.2746	0.0908	a
150.2921	0.0093	b
157.1095	70.1997	a
166.7961	42.5504	b
168.8388	0.0516	a

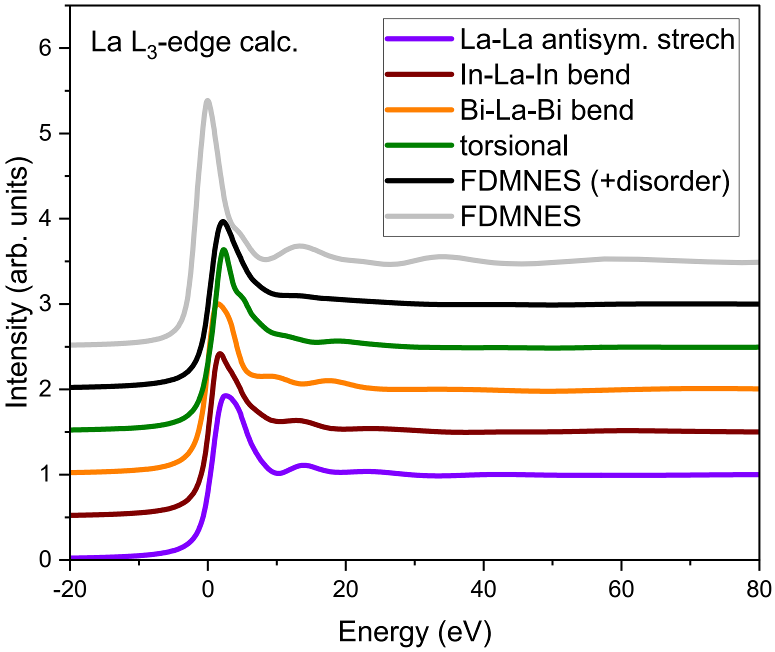

a

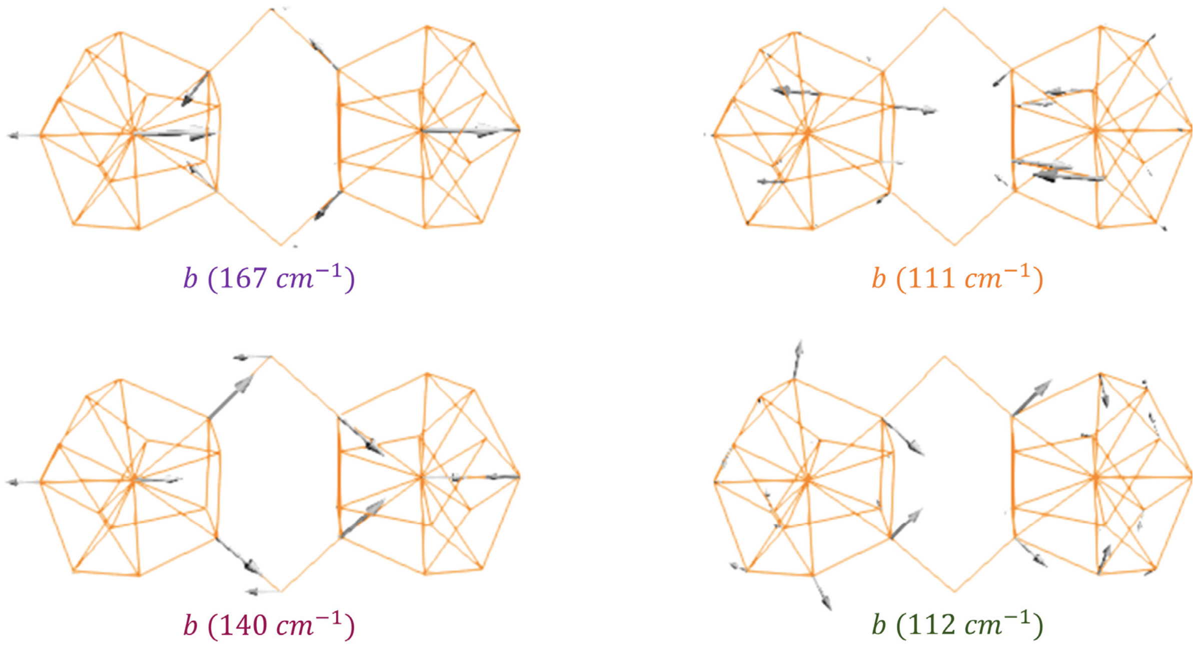

b

Figure S24. Calculated disorder effects on the La L_3-edge spectra of the lanthanide cluster $[(La@In_2Bi_{11})_2Bi_2]^{6-}$ (a). Selected vibrational modes together with their vibrational frequencies that have the largest impact on the spectral shape (b): La-La anti-symmetric stretching (b 167 cm^{-1}), In-La-In bending (b 140 cm^{-1}), Bi-La-Bi bending (b 111 cm^{-1}) and La-In-Bi-In torsional (b 108 cm^{-1}). The arrows denote the displacement vectors that are specific to each normal modes.

3 Experimental Details

General. All manipulations and reactions were performed in an argon atmosphere using standard Schlenk or glovebox techniques. Ethane-1,2-diamine (*en*) (Aldrich, 99.8 %) was dried over CaH_2, toluene (Acros Organics, 99 %) was dried over sodium. Both solvents were freshly distilled prior to use. crypt-222 (4,7,13,16,21,24-hexaoxa-1,10-diazabicyclo[8.8.8]hexacosane, Kryptofix® 222, Merck) and Lanthanum(III) oxide (99.9 %, Merck) were dried in vacuum for 13 h. The precursors $[K([2.2.2]crypt)]_2[InBi_3]$·*en* was prepared according to literature procedure.[25] The tris(tetramethylcyclopentadienyl)lanthanum $[La(C_5Me_4H)_3]$ was prepared according to literature procedure.[26,27]

Preparation of $[K([2.2.2]crypt)]_6[(La@In_2Bi_{11})(\mu\text{-}Bi)_2(La@In_2Bi_{11})]$·3*en*·3tol in a slighty modified way according to literature procedure.[28] 20 mg (12.15 µmol, 1.0 eq) $[K(crypt\text{-}222)]_2(InBi_3)$·*en* and 8 mg (15.9 mmol, 1.3 eq) $[La(CpMe_4H)_3]$ were weighed into a brown glass in the glovebox. After addition of 1.2 mL *en*, the suspension is stirred for 3 h and then filtered. The residue is discarded and the resulting greenish solution is overlayered with 2.4 mL toluene. Crystallization takes place at room temperature. After a few days, dark blue to black platelets form on the wall of the vessel. The mother liquor was discarded, the crystals were kept under toluene and harvested with a spatula covered with immersion oil.

Sample preparation. The cluster and precursor compounds were sealed in glass capillaries with a thickness of the walls of 10 µm for the synchrotron experiments described below.

SUL-X beamline at the KIT Light Source. In L_1–edge (4238 eV) XANES spectra were recorded at the SUL-X beamline of the synchrotron radiation source of the Karlsruhe Institute of Technology (KIT). SUL-X is operating with a 27 pole Wiggler as radiation source. The incoming beam was monochromized with a Si(111) fixed exit double crystal monochromator, and focused with a Kirkpatrick-Baez-mirror to about 0.1 mm vertical and 0.25 mm horizontal at sample position to consider the smaller dimension of the horizontally mounted capillaries containing the sample substances. Signals were detected in fluorescence mode with a 7 element Silicon Drift detector (Sirius, rayspec) using the $L_1M_{2,3}$ fluorescence emission for the In L_1-edge. Measurements were performed under high vacuum (10^{-5} mbar range) to minimize absorption in air of the low energy X-ray radiation.
A series of short XANES scans were done at different positions on the sealed capillaries to check for beam induced changes and to study the effect of the irradiation on the structure of the sample. The duration of a single L-edge XANES scan was about 3.5 minutes. Due to observed beam induced changes already between the first and second scan for the black (un-oxidized) 14D data have been accumulated from first scans of each scan series at different sample positions for this sample in order to get an almost non irradiation affected pristine spectrum with sufficient signal to noise ratio. For the grey (oxidized) 14D sample beam induced changes were not observed. Energies were calibrated setting the first maximum of the derivatives of In metal foil (3 µm, Goodfellow No. IN000060/6) XANES spectra to 4238 eV for the In L_1-edge. Calibration measurements were done before and after scan series. Energy shifts of sample scans were linearly interpolated between these two calibration values.
Data was processed (pre- and background correction, energy calibration, normalization to edge jump one) and evaluated using the Athena program of the IFFEFIT package and the Demeter Athena version.[29]
Measurement conditions for the Bi L_1 (16387.5 eV, PyMCA).and L_3 edges (13418.6 eV, PyMCA) were similar as for the In measurements. Bi metal foil was a 10 µm thick one from Goodfellow (No. BI000120/2).

ACT station at the CAT-ACT beamline at the KIT Light Source. La L_3-edge HR-XANES and valence band-RIXS (VB-RIXS) spectra of the La_2O_3, $[K(crypt\text{-}222)]_6[(La@In_2Bi_{11})_2Bi_2]$·3*en*·3tol and $[La(C_5Me_4H)_3]$ were collected at the KIT Light Source, Karlsruhe, Germany, using the X-ray emission

spectrometer at the ACT station of the CAT-ACT beamline.[30] Rejection of higher harmonics occurs via the optimized optics. The first, vertically collimating bare Si mirror was placed at a beam incidence of 4.4 mrad for the measured energies.[30] The DCM was equipped with a pair of Si(111) crystals. The toroidal focusing after the DCM also occurs using bare Si at the measured energies.[30] No slits were used, leading to a standard beam size of 0.5x0.5 mm^2. The X-ray emission spectrometer containing a Rowland circle with 1 m diameter in vertical focusing geometry was aligned for the La $L_{\beta2}$ emission line (5381.4 eV, 85.99° Bragg angle) and La L_3 absorption edge (5485.2 eV, 78.14° Bragg angle) using one Ge⟨422⟩ crystals with 1 m bending radius for the HR-XANES and VB-RIXS experiments, respectively. The complete spectrometer setup was installed in a He environment (~99.5 % He). The FWHM of the X-ray beam elastically scattered on a PTFE disk at 5485.2 eV was 1.8 eV. The signal was normalized by the intensity in the first ionization chamber. Measurements of HR-XANES and VB-RIXS spectra were done at room temperature. After > 15 minutes of irradiation the HR-XANES spectra started to change (cf. Figure S28). Spectra were measured for the time when no changes were observed and the process was repeated at different, fresh sample spots. The resulting spectra were averaged for analysis. The energy scale was adjusted to relative scale by subtracting the Fermi energy. Note that the powdered samples were placed in glass capillaries with 10 micrometer thickness of the wall; the samples of the clusters were as synthesized and resembled black powder.

Data analyses of La L_3-edge VB-RIXS spectra. Data was processed using the Athena program of the Demeter Package and OriginLab 2023.[29] Calibration was done setting the La L_3 edge most intense absorption peak (white line, WL) of La_2O_3 to 5491 eV. The analysis of the averaged VB-RIXS spectra continued by subtraction of a linear function from each spectrum. The linear function was determined using the background subtraction feature in the Peak Finder of OriginPro 2023's (OriginLab) Analysis tab. For this purpose, the user defined mode was used to choose low-intensity anchor points on the low and high energy ends of the spectrum and along the lowest intensity area between -10 and -28 eV (between two emission signals). This resulted in the subtraction of the linear backgrounds visible in Figure S25. The spectra with subtracted background were then read into XES Neo [31,32] and modelled using between 4 and 7 peak signals (Table S9, Figure S26). Due to the low intensity of the original spectra, the scale data button was used to enable more exact data modelling. For normalization, the background subtracted, scaled spectrum was divided by the total area of all peaks comprising the modelled 5s electron signal, resulting in comparable spectra (Figure S27). The ground laying assumption here is that the core 5s electrons have little to no participation in bonding and the signal's integral should therefore be the same for all compounds and can be normalized to one. The area of the model peaks was then divided by the same value to ascertain quantitative values for electron participation in bonding (Table S10).

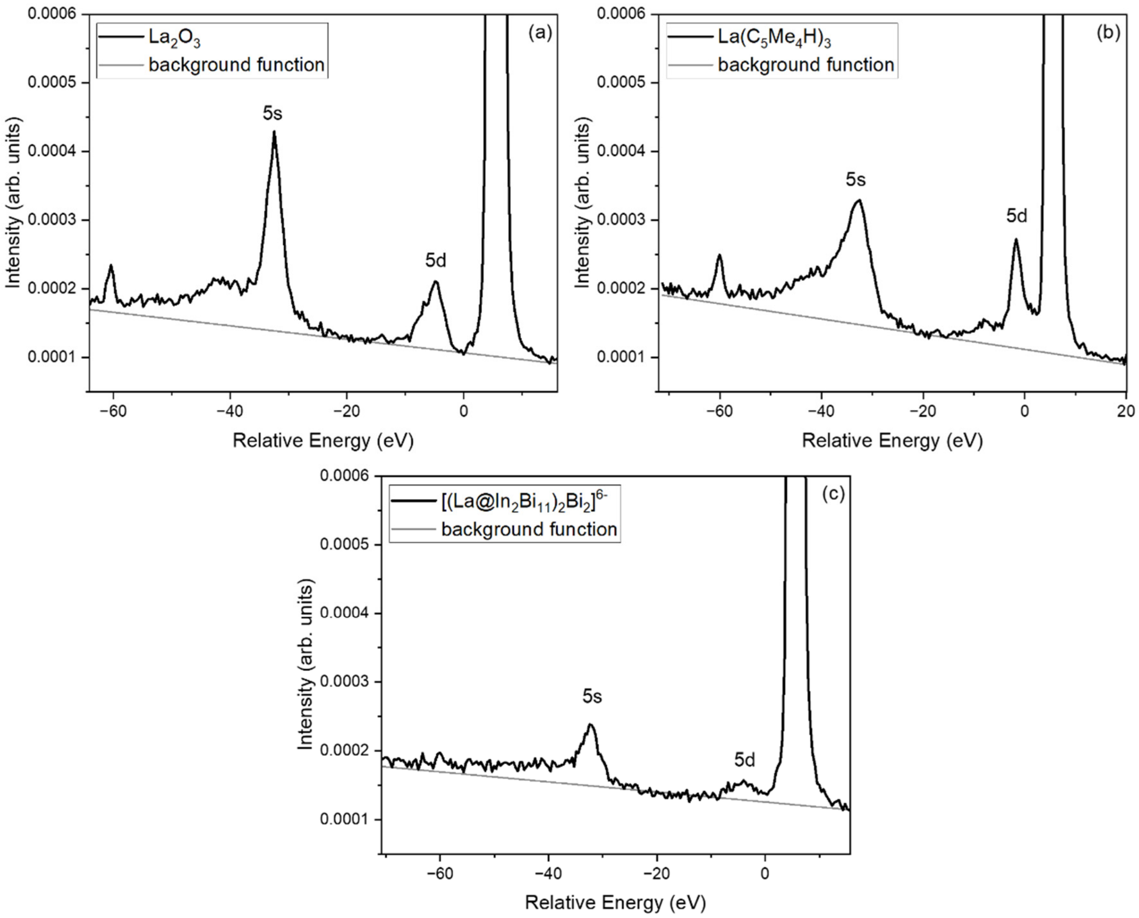

Figure S25. La L_3 edge VB-RIXS spectra as measured and the linear background functions determined using OriginPro 2023 for La_2O_3 ($y=-9.83*10^{-7}x+1.07*10^{-4}$, (a)), $[La(CMe_4H)_3]$ ($y=-1.11*10^{-6}x+1.12*10^{-4}$, (b)), and $[K(crypt\text{-}222)]_6$ $(La@In_2Bi_{11})_2Bi_2]\cdot3$*en*$\cdot$3tol ($y=-7.30*10^{-7}x+1.26*10^{-4}$, (c)). The signals corresponding to emissions from 5s core orbitals and 5d dominated molecular orbitals are marked.

Table S9. Results of the XES Neo fits for La_2O_3, $[La(CMe_4H)_3]$ and $[K(crypt\text{-}222)]_6[(La@In_2Bi_{11})_2Bi_2]\cdot3$*en*$\cdot$3tol. Because Voigt was chosen as a peak type for all peaks, both the variables sigma (as the variable for the Gaussian part of the Voigt profile) and gamma (as the variable for the Lorentzian part of the Voigt profile) are relevant. Values are given with three significant figures, with exceptions in cases where less are outputted.

Compound	Bkg	Peak	Type	Rel. Energy [eV]	Sigma	Gamma	Amplitude	Area
La_2O_3	-0.1	1	Voigt	5.93±0.01	0.63±0.01	0.15±0	1.97E4±26.5	3.48E4±745
		2	Voigt	-4.64±0.08	0.83±0.06	0.79±0.07	89.0±4.32	260±0.79
		3	Voigt	-7.41±0.12	0.77±0.12	0.82±0.09	26.0±2.9	73.8±0.48
		4	Voigt	-32.3±0.12	1.04±0.13	0.95±0.12	271±3.85	974±1.51
		5	Voigt	-40.5±0.12	4.87±0.11	5.98±0.04	52.0±4.38	919±6.8E-2
		6	Voigt	-60.3±0.09	0.57±0.06	0.58±0.06	57.5±1.84	118±0.86
$[(La@In_2Bi_{11})_2Bi_2]^{6-}$	0	1	Voigt	5.5±0.01	0.53±0.01	0.16±0.0	2.52E4±13.4	3.66E4±897
		2	Voigt	-4.76±0.41	1.25±0.13	1.84±0.11	21.3±0.65	109±5.4E-2
		3	Voigt	-32.4±0.14	1.19±0.12	1.13±0.12	74.3±4.39	308.5±0.16
		4	Voigt	-40.1±0.11	6.90±0.1	6.76±0.11	27.8±1.77	644±0.30
		5	Voigt	-60.1±0.04	0.67±0.07	0.79±0.08	61.8±0.27	61.8±0.27
$[La(C_5Me_4H)_3]$	59.72087	1	Voigt	5.56±0.0	0.66±0.0	0.15±0.0	2.06E5±163	3.66E5±6.44E3
		2	Voigt	-1.55±0.11	0.68±0.02	1.21±0.04	1.88E3±52.1	5.79E3±20.8
		3	Voigt	-7.5±0.03	1.53±0.07	1.33±0.13	275±5.56	1.43E3±0.50
		4	Voigt	-32.65±0.01	1.84±0.22	1.53±0.06	1.96E3±3.85	1.21E4±1.3
		5	Voigt	-40.82±0.26	3.56±0.16	4.82±0.25	731±6.92	9.99E3±4.31
		6	Voigt	-60.16±0.0	0.66±0.01	0.37±0.01	726±5.49	1.44E3±7.06
		7	Voigt	-36.56±0.08	0.95±0.05	0.77±0.12	450±69.8	1.43E3±0.85

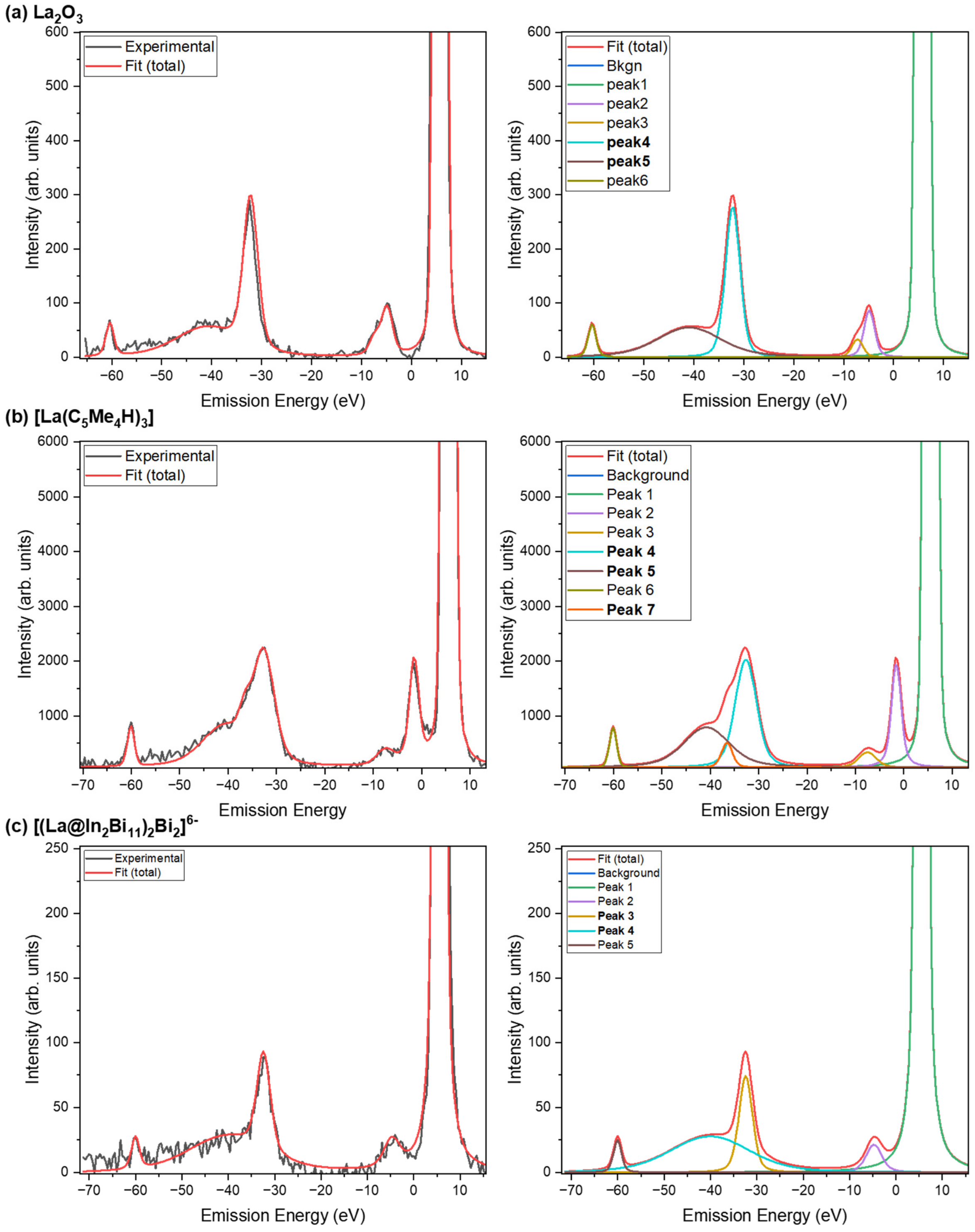

Figure S26. La L_3 edge VB-RIXS spectra with fit resulting from XES Neo fits (Table S9) and the peaks comprising those fits for La_2O_3 (a), $[La(CMe_4H)_3]$ (b), and $[K(crypt\text{-}222)]_6[(La@In_2Bi_{11})_2Bi_2]\cdot 3$*en*$\cdot$3tol (c). Peaks belonging to the 5s signal, which was used for normalization, are bolded.

Table S10. Areas of the fit peaks for La_2O_3, [K(crypt-222)]$_6$[(La@In_2Bi_{11})$_2Bi_2$]·3*en*·3tol and [La(CMe_4H)$_3$] unnormalized and normalized by the total fitted area of the s signal. Values are given with three significant figures, with exceptions in cases where less are outputted. The numbering of the peaks corelates with the numbering in Figure S26. The peaks relevant for the 5d electron contributions are marked in bold. The total normalized 5d contributions for the three compounds are as follows: 0.18 (La_2O_3), 0.11 ([K(crypt-222)]$_6$[(La@In_2Bi_{11})$_2Bi_2$]·3*en*·3tol) and 0.31 ([La(CMe_4H)$_3$]).

Compound	Total Area of s Signal	Peak	Area (not normalized)	Area (normalized)
La_2O_3	1.89E3	1	3.48E4±745	18.4±0.39
		**2[b]**	**260±0.789**	**0.14±4.17E-4**
		**3[b]**	**73.8±0.48**	**3.90±E-2±2.54E-4**
		4[a]	974±1.51	0.51±7.95E-4
		5[a]	919±6.8E-2	0.49±3.59E-5
		6	118±0.86	6.23E-2±4.55E-4
[(La@In_2Bi_{11})$_2Bi_2$]$^{6-}$	952	1	3.66E4±897	38.4±0.94
		**2[b]**	**109±5.4E-2**	**0.11±5.67E-5**
		3[a]	308.5±0.16	0.32±1.63E-4
		4[a]	644±0.30	0.68±313E-4
		5	61.8±0.27	6.48E-2±2.82E-4
[La(C_5Me_4H)$_3$]	2.36E4	1	3.66E5±6.44E3	15.2±0.27
		**2[b]**	**5.79E3±20.8**	**0.25±8.82E-4**
		**3[b]**	**1.43E3±0.50**	**6.08E-2±2.10E-5**
		4[a]	1.21E4±1.3	0.52±5.52E-5
		5[a]	9.99E3±4.31	0.42±1.83E-4
		6	1.44E3±7.06	6.13E-2±3.00E-4
		7[a]	1.43E3±0.85	6.06E-2±3.60E-5

[a] Part of the 5s peak [b] Part of the 5d peak

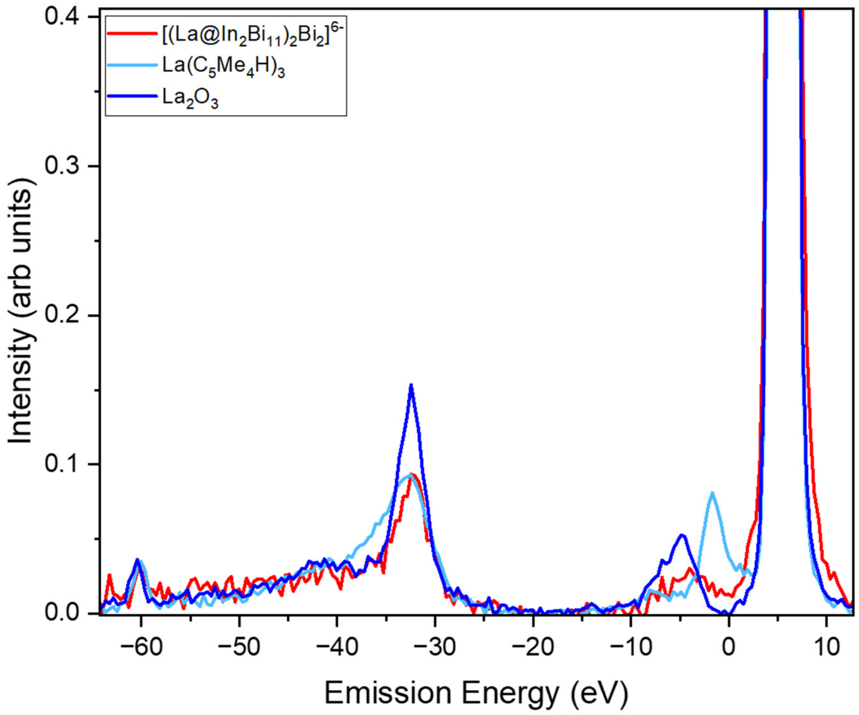

Figure S27. VB-RIXS spectra of La_2O_3, [La(CMe_4H)$_3$], and [K(crypt-222)]$_6$[(La@In_2Bi_{11})$_2Bi_2$]·3*en*·3tol normalized by the area under the s peak.

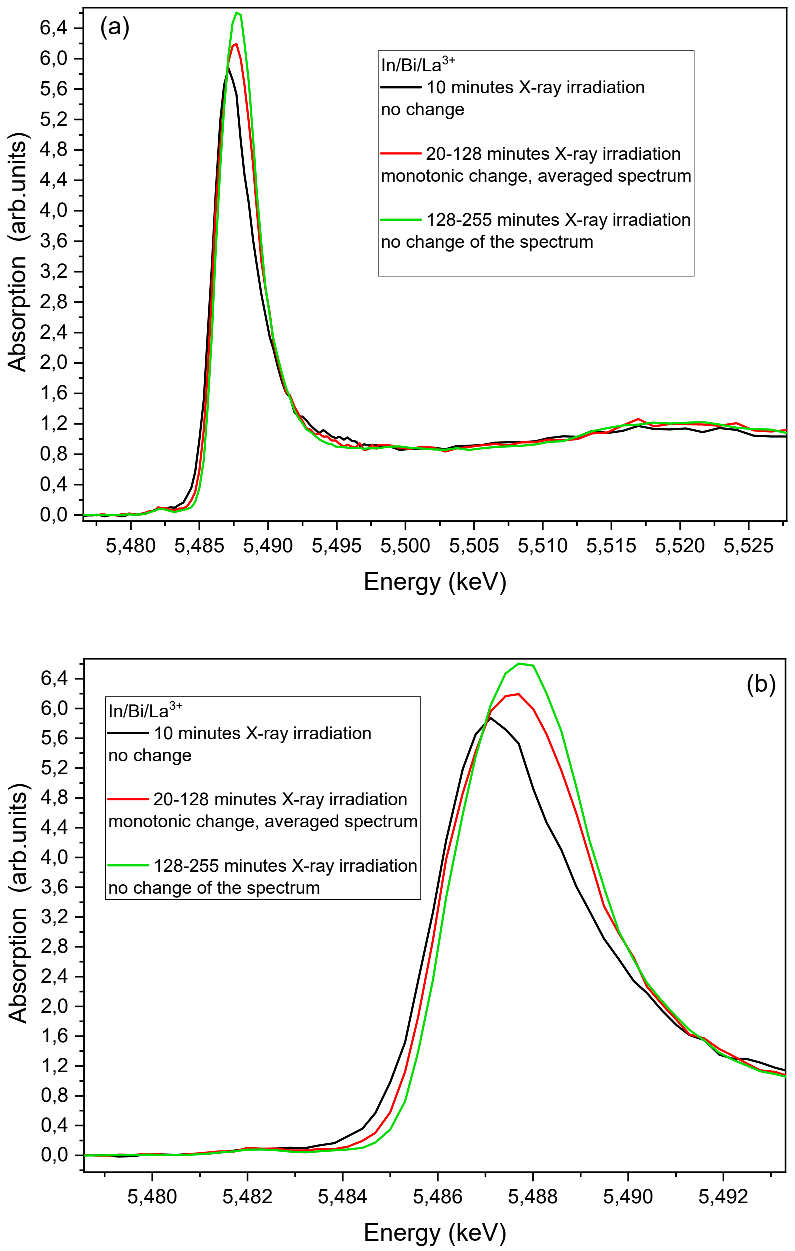

Figure S28. La L_3-edge HR-XANES spectra of $[K(crypt\text{-}222)]_6[(La@In_2Bi_{11})_2Bi_2]\cdot 3en\cdot 3tol$ measured as a function of time at the ACT station of the CAT-ACT beamline. Note that the spectra are averaged over specific time intervals as indicated in (b), and they are neither normalized nor calibrated. The complete spectra are shown in (a), while a zoom into the initial portion of the spectra is presented in (b). The measurements were performed consecutively on the same sample spot.

INE-Beamline at the KIT Light Source. Bi L_3-edge XANES of $[K(crypt\text{-}222)]_6[(La@In_2Bi_{11})_2Bi_2]\cdot 3\textit{en}\cdot 3tol$ and its oxidized pendant were collected at the INE-Beamline of the KIT Light Source, Karlsruhe, Germany using a fluorescence mode set up.[33] Rejection of higher harmonics at the INE beamline is sufficient up until the Si K-edge using an indirectly water-cooled Rh coated collimating Si mirror, a Lemmonier-type DCM and a Rh coated toroidal Si mirror.[33] The DCM was equipped with a pair of Ge(422) crystals. Bi Lα characteristic fluorescence was measured using a combination of a Vortex 4 Element SDD and a Vortex 1 Element SDD detector. The ROI was set to 10640–11040 eV. The signal was normalized by the intensity in the first ionization chamber, before the rest of the experimental set-up. Data was processed using the Demeter Athena Package and OriginLab 2024. The calibration of the data was done using a Pt reference foil; the energy of the Pt L_2 edge was set to 13273 eV.

ID26 beamline at the ESRF. La L_3-edge HR-XANES spectra were measured using the X-ray emission spectrometer in focusing vertical Rowland circle geometry with an avalanche photodiode, silicone drift diode detector in the ID26 beamline at ESRF, Grenoble, France.[34] The beamline optics are optimized for higher harmonic rejection.[35] It relies on a water-cooled Si deflecting mirror (typically between 1.5 and 5 mrad grazing angle) and a Kirkpatrick-Baez mirror set dynamic focusing (between 2 and 10 mrad grazing angle for each mirror, respectively).[36] All three mirrors have Cr and Pt metallic strips in order to enhance energy cutoff and reflectivity.[36] The spectrometer was aligned for the La $L\alpha_1$ emission line (4651 eV, 78.27° Bragg angle) and La L_3 absorption edge (5482.7 eV, 79.02° Bragg angle) using one Si⟨400⟩ crystal with 1 m bending radius for the HR-XANES measurements. A 1000x200 μm^2 slit was used, resulting in a beam size of ca 500x200 μm^2 (horizontal x vertical size), based on the maximum horizontal beam size at ID26.[37] The signal was normalized by the intensity in the first ionization chamber. La L_3-edge HR-XANES spectra of samples were measured between 5470 and 5540 eV with a 0.2 eV step size by continuously scanning the energy of the incident beam. 30 repeated measurements were collected for 30 s per spectrum on the same sample spot. The FWHM of the elastically scattered X-ray beam at 5489 eV was 0.5 eV. The spectrum of the cluster started changing after 60 s total irradiation time on one spot. Spectra were calibrated using the L_3 absorption edge of the La_2O_3 spectrum, which had previously been determined via measurement and alignment to the V K-edge at the SUL-X beamline at the KIT Light Source.

4 References

[1] TURBOMOLE V7.8 2023, a development of University of Karlsruhe and Forschungszentrum Karlsruhe GmbH, 1989-2007, TURBOMOLE GmbH, since 2007; available from https://www.turbomole.org.
[2] S. G. Balasubramani, G. P. Chen, S. Coriani, M. Diedenhofen, M. S. Frank, Y. J. Franzke, F. Furche, R. Grotjahn, M. E. Harding, C. Hättig, A. Hellweg, B. Helmich-Paris, C. Holzer, U. Huniar, M. Kaupp, A. Marefat Khah, S. Karbalaei Khani, T. Müller, F. Mack, B. D. Nguyen, S. M. Parker, E. Perlt, D. Rappoport, K. Reiter, S. Roy, M. Rückert, G. Schmitz, M. Sierka, E. Tapavicza, D. P. Tew, C. van Wüllen, V. K. Voora, F. Weigend, A. Wodyński, J. M. Yu, *J. Chem. Phys.*. **2020**, *152*, 184107.
[3] A. Klamt, G. Schüürmann, *J. Chem. Soc., Perkin Trans*. **1993**, 2. 799-805.
[4] A. Pausch, *J. Chem. Theor. Comput.* **2024**, *20*, 3169-3183.
[5] J. P. Perdew, M. Ernzerhof, K. Burke, *J. Chem. Phys.* **1996**, *105*, 9982-9985.
[6] C. Adamo, V. Barone, *J. Chem. Phys.* **1999**, *110*, 6158-6170.
[7] P. Pollak, F. Weigend, *J. Chem. Theor. Comput.* **2017**, *13*, 3696-3705.
[8] K. Krause, M. E. Harding, W. Klopper, *Mol. Phys.* **2015**, *113*, 1952-1960.
[9] L. Monzel, C. Holzer, W. Klopper, *J. Chem. Phys.* **2023**, *158*, 144102.
[10] X. Gui, C. Holzer, W. Klopper, *J. Chem. Theor. Comput.* **2018**, *14*, 2127-2136.
[11] D. Peng, N. Middendorf, F. Weigend, M. Reiher, *J. Chem. Phys.* **2013**, *138*, 184105.
[12] Y. J. Franzke, N. Middendorf, F. Weigend, *J. Chem. Phys.* **2018**, *148*, 104110.
[13] Y. J. Franzke, R. Treß, T. M. Pazdera, F. Weigend, *Phys. Chem. Chem. Phys.* **2019**, *21*, 16658-16664.
[14] C. Holzer, W. Klopper, *J. Chem. Phys*. **2019**, *150*, 204116.
[15] M. Kehry, W. Klopper, C. Holzer, *J. Chem. Phys.* **2023**, *159*, 044116.
[16] M. E. Harding, private communication, **2025**.
[17] K. Krause, W. Klopper, *J. Comput. Chem.* **2017**, *38*, 383-388.
[18] L. S. Cederbaum, W. Domcke, J. Schirmer, *Phys. Rev. A* **1980**, *22*, 206-222.
[19] A. Barth, L. S. Cederbaum, *Phys. Rev. A* **1981**, *23*, 1038-1061.
[20] S. Bernadotte, A. J. Atkins, C. R. Jacob, *J. Chem. Phys.* **2012**, *137*, 204106.
[21] M. K. Kehry, PhD thesis, Karlsruher Institut für Technologie (DE), **2022**.
[22] O. Bunău, A. Y. Ramos, Y. Joly, *Int. Tables Crystallogr. I*, **2024**, *I*, 752-757.
[23] Y. Joly, A. Ramos, O. Bunău, *Int. Tables Crystallogr. I*, **2024**, *I*, 114–120.
[24] E. J. Baerends, N. F. Aguirre, J. Autschbach, F. M. Bickelhaupt, R. Bulo, C. Cappelli, A. C. T. van Duin, F. Egidi, C. Fonseca Guerra, A. Förster, M. Franchini, T. P. M. Goumans, T. Heine, M. Hellström, C. R. Jacob, L. Jensen, M. Krykunov, E. Van Lenthe, A. Michalak, M. M. Motoraj, J. Neugebauer, V. P. Nicu, P. Philipsen, H. Ramanantoanina, R. Rüger, G. Schreckenbach, M. Stener, M. Swart, J. M. Thijssen, T. Trnka, L. Visscher, A. Yakovlev, S. van Gisbergen, *J. Chem. Phys.* **2025**, *162*, 162501.
[25] L. Xu, S. C. Sevov, *Inorg. Chem.* **2000**, *39*, 5383-5389.
[26] H. Schumann, M. Glanz, H. Hemling, F. E. Hahn, Z. *Anorg. Allg. Chem.* **1995**, *621*, 341-345.
[27] H. Schumann, M. Glanz, H. Hemling, *J. Organomet. Chem.* **1993**, *445*, C1-C3.
[28] B. Weinert, F. Weigend, S. Dehnen, *Chem. Eur. J.* **2012**, *18*, 13589-13595.
[29] B. Ravel, M. Newville, *J. Synchrotron Radiat.* **2005**, *12*, 537-541.
[30] A. Zimina, K. Dardenne, M. A. Denecke, D. E. Doronkin, E. Huttel, H. Lichtenberg, S. Mangold, T. Pruessmann, J. Rothe, T. Spangenberg, R. Steininger, T. Vitova, H. Geckeis, J. D. Grunwaldt, *Rev. Sci. Instrum.* **2017**, *88*, 113113.
[31] A. Humiston, M. L. Lau, T. Stack, E. Restuccia, A. Herrera-Gomez, M. Long, D. T. Olive, J. Terry, *J. Vac. Sci. Technol. A* **2025**, *43*, 043411.
[32] XES Neo, Open source Repository For the XES Neo Analysis Package, version 0.0.6, can be found under https://github.com/lanl/XES_Neo_Public, **2025** (accessed 15.07.2025).
[33] J. Rothe, S. Butorin, K. Dardenne, M. A. Denecke, B. Kienzler, M. Löble, V. Metz, A. Seibert, M. Steppert, T. Vitova, C. Walther, H. Geckeis, *Rev. Sci. Instrum.* **2012**, *83*, 043105.
[34] P. Glatzel, A. Harris, P. Marion, M. Sikora, T.-C. Weng, C. Guilloud, S. Lafuerza, M. Rovezzi, B. Detlefs, L. Ducotte, *J. Synchrotron Radiat*. **2021**, *28*, 362-371.
[35] C. Gauthier, V. A. Sole, R. Signorato, J. Goulon, E. Moguiline, *J. Synchrotron Radiat*. **1999**, *6*, 164-166.
[36] R. Signorato, V. A. Sole, C. Gauthier, *J. Synchrotron Radiat.* **1999**, *6*, 176-178.
[37] European Synchrotron Radiation Facility, ID26 - X-ray Absorption and Emission Spectroscopy, can be found under https://www.esrf.fr/UsersAndScience/Experiments/EMD/ID26, **2025** (accessed 23.04.2025).
